# The impact of SO_2_ on wine flavanols and indoles in relation to wine style and age

**DOI:** 10.1038/s41598-018-19185-5

**Published:** 2018-01-16

**Authors:** Panagiotis Arapitsas, Graziano Guella, Fulvio Mattivi

**Affiliations:** 1 Department of Food Quality and Nutrition, Research and Innovation Centre, Fondazione Edmund Mach (FEM), San Michele all’Adige, Italy; 2 Centre for Agriculture, Food and the Environment, University of Trento, San Michele all’Adige, Italy; 3 Bioorganic Chemistry Laboratory, Department of Physics, University of Trento, Trento, Italy

## Abstract

Wine has one of the broadest chemical profiles, and the common oenological practice of adding the antioxidant and antimicrobial sulfur dioxide has a major impact on its metabolomic fingerprint. In this study, we investigated novel discovered oenological reactions primarily occurring between wine metabolites and sulfur dioxide. The sulfonated derivatives of epicatechin, procyanidin B2, indole acetic acid, indole lactic acid and tryptophol were synthesized and for the first time quantified in wine. Analysis of 32 metabolites in 195 commercial wines (1986–2016 vintages) suggested that sulfonation of tryptophan metabolites characterised white wines, in contrast to red wines, where sulfonation of flavanols was preferred. The chemical profile of the oldest wines was strongly characterised by sulfonated flavanols and indoles, indicating that could be fundamental metabolites in explaining quality in both red and white aged wines. These findings offer new prospects for more precise use of sulfur dioxide in winemaking.

## Introduction

Sulfur dioxide (SO_2_) is the most widely used additive in winemaking. Due to its antimicrobial action and antioxidant effect, SO_2_ is able to protect wine from various unwanted reactions and is thus widely considered as an indispensable additive in winemaking^1^. At the same time, SO_2_ and sulfites are among food allergens, since they may cause breathing difficulty, sneezing, hives, migraine and other problems^2^. The legal limit for total SO_2_ concentration according to the OIV is 150–200 mg/L for dry wines, while in exceptional cases it can reach up to 400 mg/L^3^ for some sweet wines.

Sulfonation, the addition of a sulfonic acid group (-SO_3_H) to an organic compound, is a widespread industrial process used in a diverse range of products, including dyes and colour intensifiers, pigments, medicines, detergent additives, surfactants in laundry, pesticides and organic intermediates^4^. Moreover, sulfonation has a major function in modulating the biological activities of a wide number of chemicals, such as drugs, toxic chemicals, hormones and neurotransmitters^5,6^. This reaction is also known to occur in wine, involving several metabolites, such as polyphenols and indoles^1,7–10^.

**Figure 1:**
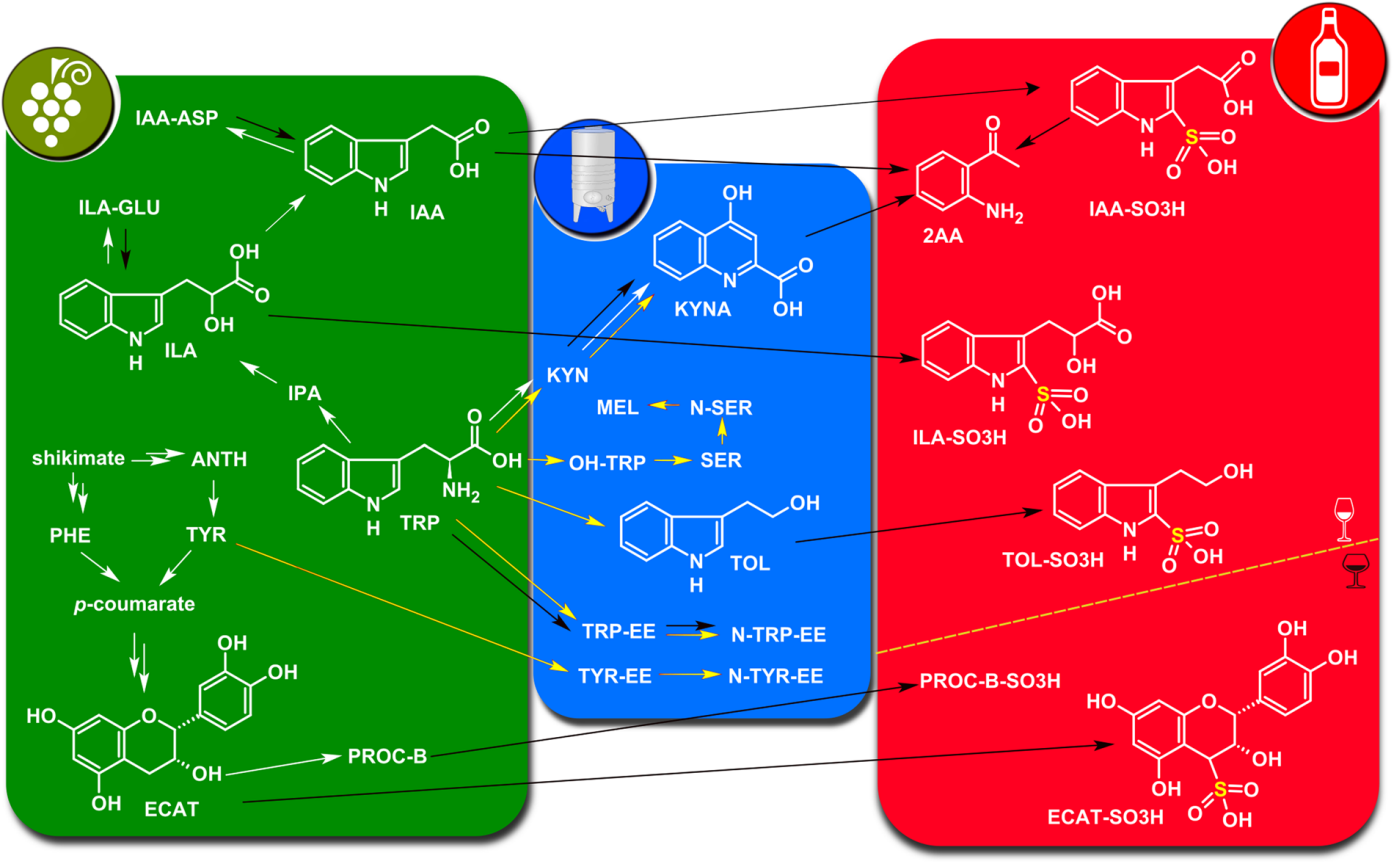
Diagram of the biological and chemical reactions presented in the work. The green panel and white arrows on the left are dedicated to plant metabolism, the central blue panel and yellow arrows to yeast metabolism and the red panel and black arrows on the right to wine ageing. ILA: indole 3-lactic acid; GLU: glucose; IAA: indole 3-acetic acid; IPA: indole 3-pyruvic acid; TRP: tryptophan; TRP-EE: tryptophan ethyl ester; N-TRP-EE: N-acetyl-tryptophan ethyl ester; KYNA: kynurenic acid; KYN: kynurenine; TOL: tryptophol; TYR: tyrosine; TYR-EE: tyrosine ethyl ester; N-TYR-EE: N-acetyl-tyrosine ethyl ester; ECAT: epicatechin; PRO-B: type B procyanidin B. The grape cluster, the wine bottle and the wine glass icons were made by Freepik from www.flaticon.com. The cuve inox was made by Olivier Colas (http://olouf.fr) (Own work) [CC BY-SA 4.0 (https://creativecommons.org/licenses/by-sa/4.0)].

Phenolic metabolites known as condensed tannins (i.e. procyanidins and prodelphinidins) are oligomers consisting of several flavan-3-ol units, such as catechin (CAT) and epicatechin (ECAT), gallocatechin and epigallocatechin, and epicatechin gallate^11^ (Fig. 1). Condensed tannins are important components in wine quality, since they contribute to mouthfeel, long-term colour stability, chemical stability to oxidation and the nutritional value of wine^1,12^. Condensed tannins in wine derive primarily from the seeds and skin of grapes during winemaking, so red wines contain a larger amount than white wines, but they can also be the result of oenological practices^1^. Foo *et al*.^13^ studied the reactivity of type B procyanidins (PROC-B) with sodium hydrogen sulfite, and proved that this reaction leads to the formation of 4β-sulfonates of both epicatechin and oligomeric procyanidins (Fig. 1). Recently, sulfonated dimeric and monomeric flavanols were detected in red wine and were suggested as promising tentative markers of sub-optimal wine storage^10,14^.

On the other hand, tryptophan (TRP) is an essential amino acid, and its metabolism is vital for many biological functions in plants and animals. Tryptophan catabolites include the best known plant auxin class hormone indole acetic acid (IAA)^15^, the neurotransmitter serotonin (SER)^16^, the neurohormone melatonin (MEL)^16^, the antiexcitotoxic and anticonvulsant kynurenic acid (KYNA)^17,18^, the sleep regulator and yeast quorum-sensing tryptophol (TOL)^19,20^ and indole lactic acid (ILA). In 1962, Thesing *et al*.^21^ described the sulfonation of indole, and 50 years later sulfonated tryptophol (TOL-SO_3_H) and the sulfonated indole lactic acid glucoside (ILA-GLU-SO_3_H) were detected in wine^9^. Hoeniche *et al*.^22^ suggested that sulfonation of IAA is an intermediate step for the production of 2-aminoacetophenone (2AA), which is responsible for the wine ‘off-flavour’ called untypical ageing (Fig. 1).

In our previous studies, the use of an untargeted metabolomics protocol allowed us to discover sulfonated flavanols in red wines, positively correlated with wine ageing and storage^10,14^, and sulfonated indoles in white wines, positively correlated with packaging oxygen^9^. These projects tried to study wine ageing through a fully controlled experimental design, while the reactions discovered were also confirmed with wine model solution experiments. Moreover, we discussed the influence of (total and free) SO_2_ concentration, oxygen, temperature, pH and the wine cultivar. With this project, we decided to go a step further, trying to validate our previous hypothesis with a wide variability of commercial samples produced by different wineries for purely commercial reasons and not for laboratory use. None of the above-mentioned sulfonated metabolites are commercially available and their concentration range in wine is still unknown. Therefore, the goals of this work were (i) the preparation of standards for sulfonated indolic and flavanic compounds, (ii) the development and validation of an analytical LC-MS method capable of quantifying them in wine, and (iii) surveying of a large number of wines in order to quantify their presence in different types/vintages of wines.

## Results and Discussion

The protocol used was based on reverse phase separation with ultra-high-pressure liquid-chromatography (UHPLC), coupled to a tandem mass spectrometer (MS) operating in MRM (Multiple Reaction Monitoring) mode, which is currently the gold standard for accurate detection of trace compounds. Indeed, the reverse phase LC-MS technique is particularly suitable for analysing the indolic metabolites of tryptophan metabolism and polyphenols^23–29^. This study quantified 20 tryptophan-related metabolites, 3 tyrosine-related metabolites, phenylalanine, abscisic acid and its glucoside, and 6 flavanol-related metabolites in wine (Fig. 1 and Supplementary Table S1). The validation parameters, such as the calibration curve data (limit of quantification and linearity) for each metabolite, were comparable with cited published methods^23–27^, and the matrix effects were also analogous to earlier published data for wine, must and fermented products^18,23,24^ (Supplementary Table S1). Moreover, Supplementary Table S1 reports the LC-MS parameters, such as the retention times and quantifier/qualifier MS transitions. The use of quality control^30^ (QC) samples, which were analysed every 10 real samples, determined a good instrumental variability during the analysis (Supplementary Table S2). The relative coefficient of variation (%CV) of each analyte was measured separately for a red wine (QC_R_), white wine (QC_W_) and sparkling wine (QC_S_) pooled QC sample. The results were comparable with our previous experience^9,14,30,31^, where higher %CV values were registered for analytes closer to the limit of detection (Supplementary Table S2).

For non-commercially available metabolites, the simple semi-synthesis protocol described in the methods section was used to produce C-sulfonated analogues of IAA, ILA, and TOL, thus IAA-SO_3_H, ILA-SO_3_H, and TOL-SO_3_H correspondingly (Fig. 1). All three sulfonations occur at the same carbon position of the indole scaffold, establishing a C-S link, as compared to the O-S link of enzymatic sulfonations^5,32^. To our knowledge, ILA-SO_3_H and TOL-SO_3_H are described here for the first time in the literature. The ^1^H, ^13^C and two-dimensional NMR spectra of novel compounds are reported in Supplementary Figures S1–S12. Sulfonated epicatechin (ECAT-SO_3_H) and sulfonated procyanidin B2 (PROC-B-SO_3_H) were prepared in accordance with our previous works^10,13^, proving the high stereo-selectivity of the reaction, which requires a terminal epicatechin unit and produces a C4 adduct with 4β stereochemistry^10^.

**Figure 2:**
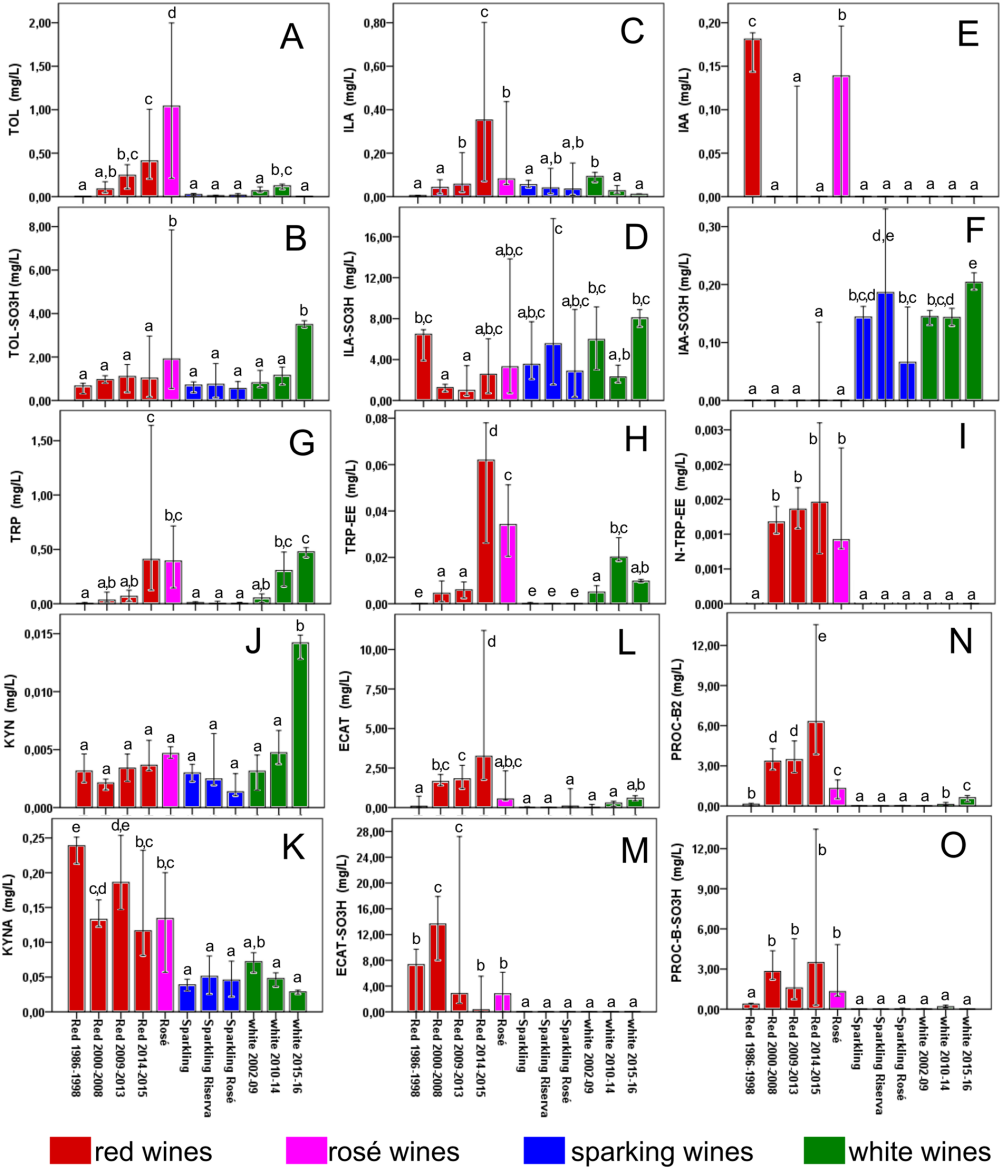
Metabolite mean concentration for the various groups of wine. The error bars represent a 95% confidence interval, ranging in number from 12 to 126 (Supplementary Table S3). Means not sharing a letter are significantly different (Supplementary Table S4).

**Figure 3:**
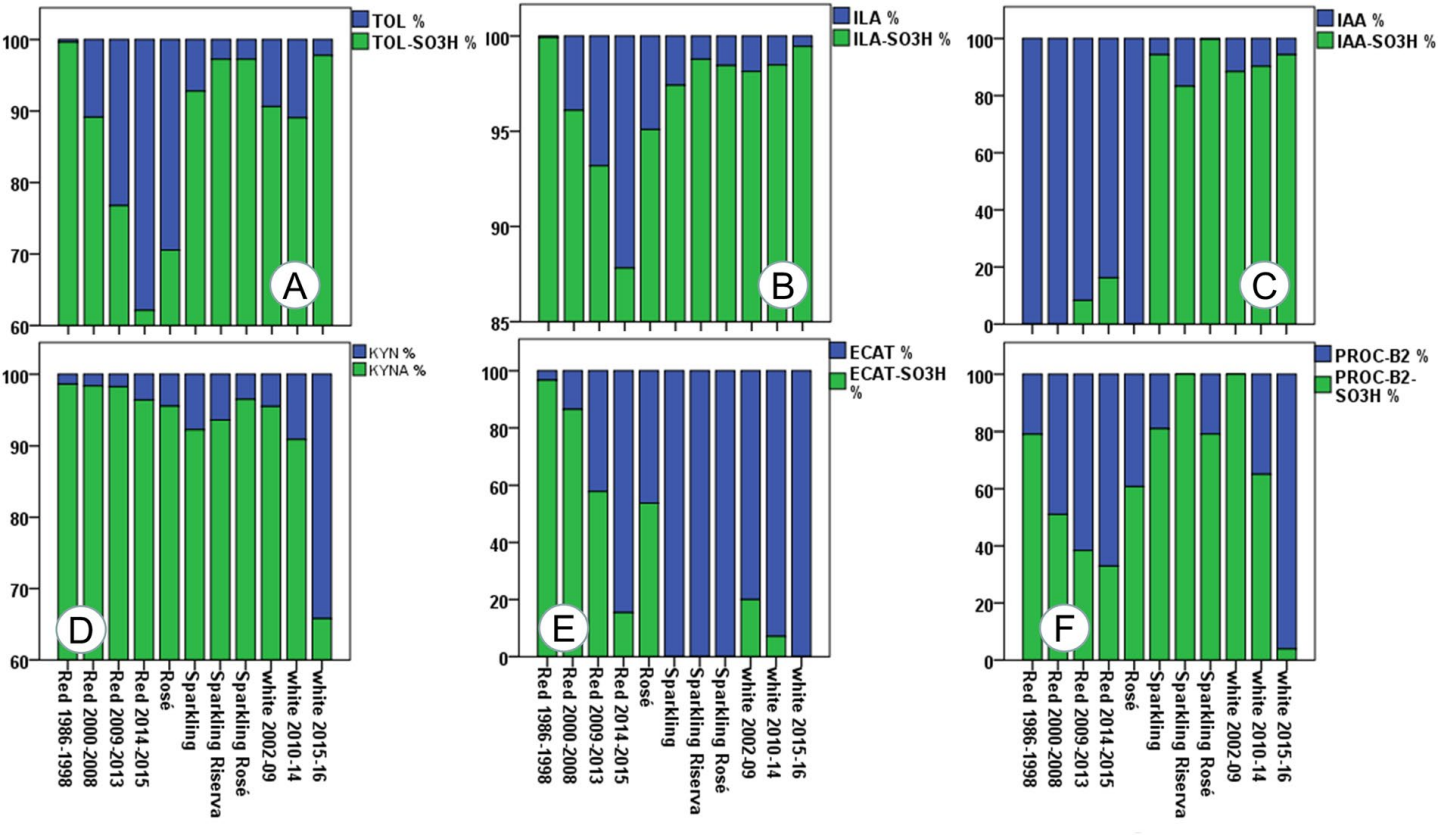
Comparison of the relative percentage concentration for six sets of metabolites in relation to the various groups of wine. (**A**) Tryptophol set: TOL/TOL-SO_3_H; (**B**) Indole lactic acid set: ILA/ILA-SO_3_H; (**C**) Indole acetic acid set: IAA/IAA-SO_3_H; (**D**) Kynurenic acid set: KYN/KYNA; (**E**) Monomeric flavanol set: ECAT/ECAT-SO_3_H; (**E**) Procyanidin type B2 set: PROC-B2/PROC-B-SO_3_H.

**Figure 4:**
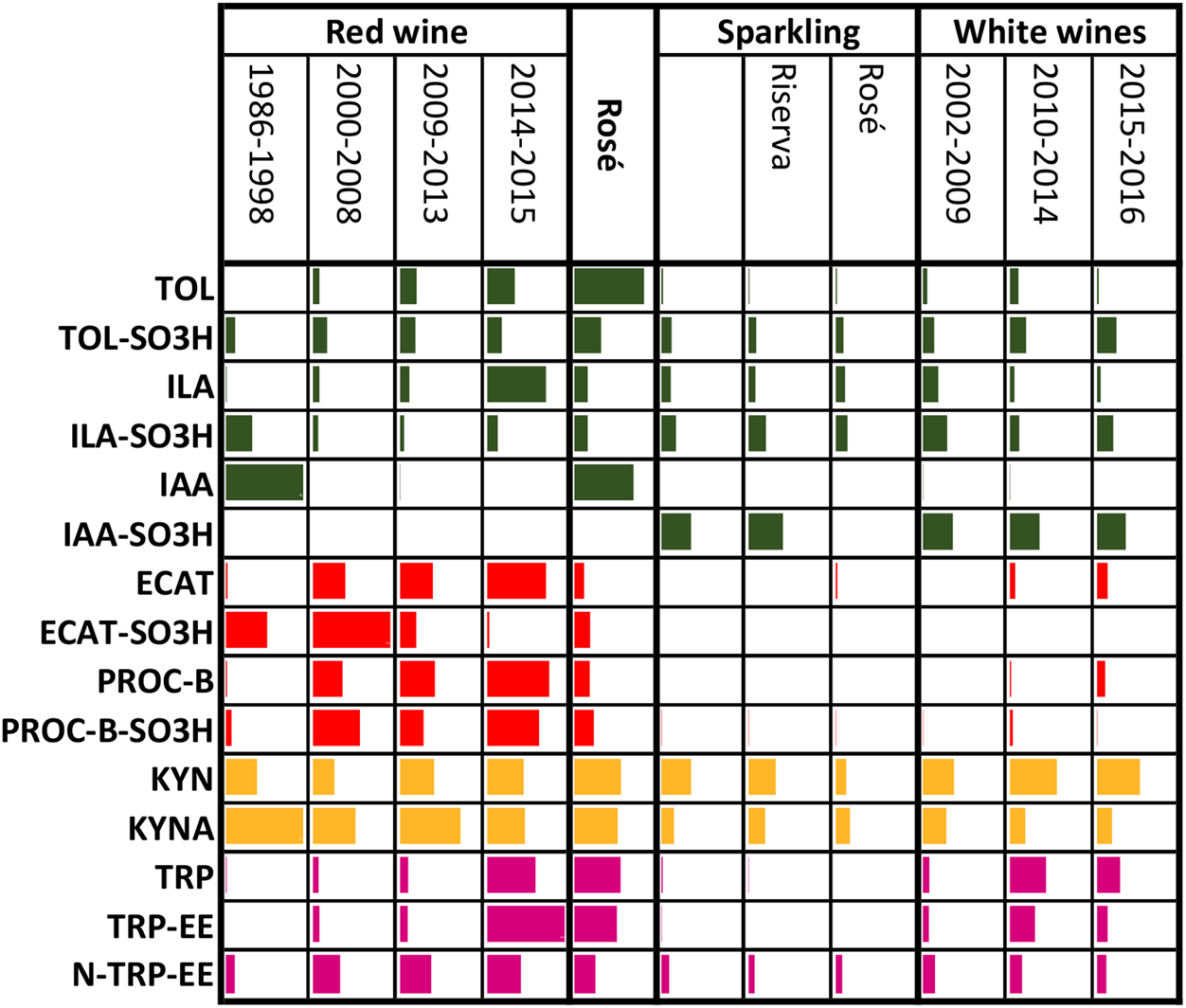
Data bar graph with the trend for the principal metabolites in the various wine groups (green: free/sulfonated indoles; red: free/sulfonated flavanols; orange: KYN/KYNA; purple: tryptophan esters). ILA: indole 3-lactic acid; GLU: glucose; IAA: indole 3-acetic acid; IPA: indole 3-pyruvic acid; TRP: tryptophan; TRP-EE: tryptophan ethyl ester; N-TRP-EE: N-acetyl-tryptophan ethyl ester; KYNA: kynurenic acid; KYN: kynurenine; TOL: tryptophol; TYR: tyrosine; TYR-EE: tyrosine ethyl ester; N-TYR-EE: N-acetyl-tyrosine ethyl ester; ECAT: epicatechin; PRO-B: procyanidin B2 (Supplementary Fig. S13). Longer bars represent higher relative values.

**Figure 5:**
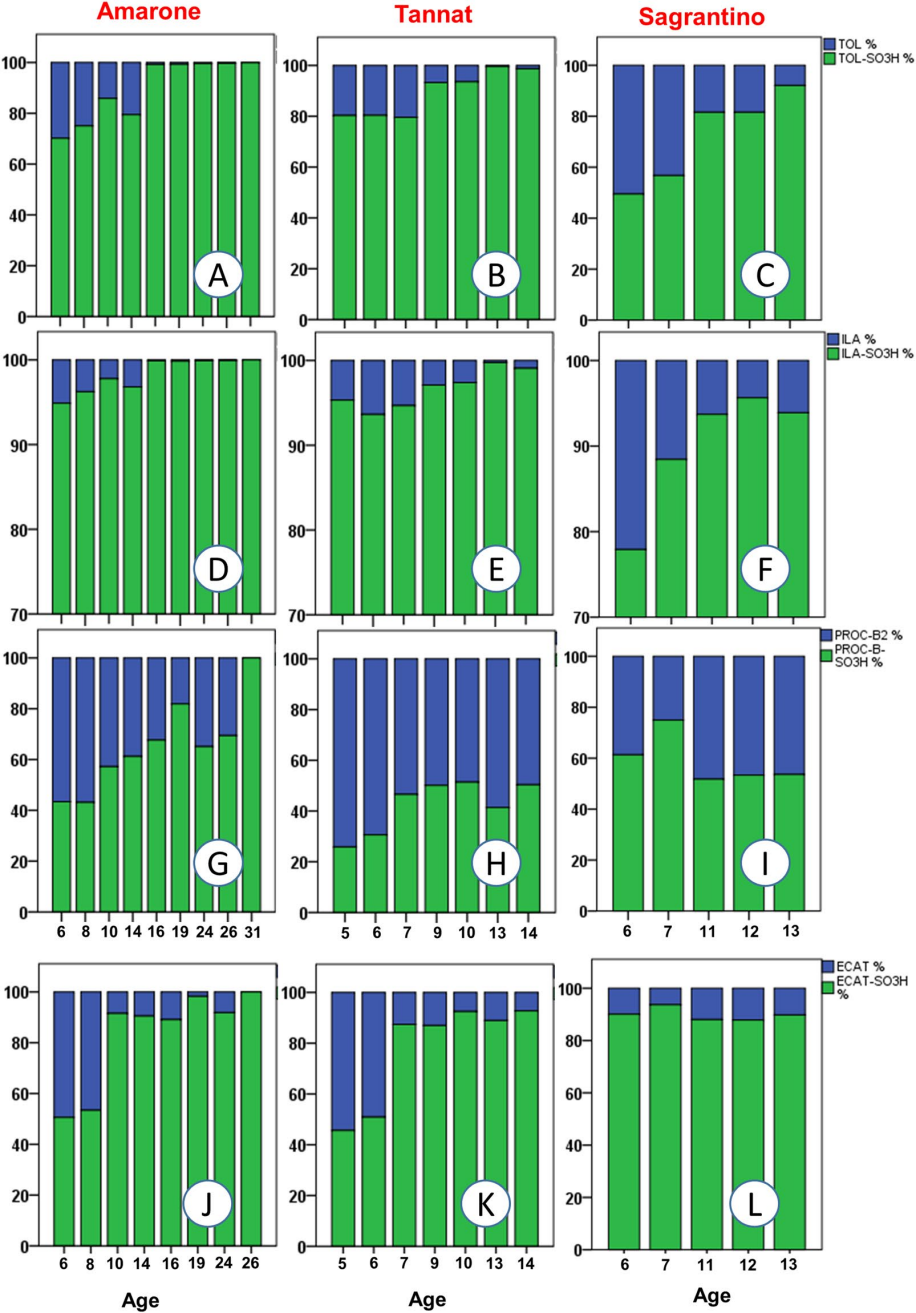
Comparison of the relative percentage concentration for four sets of metabolites in relation to the wine age of Amarone (**A**,**D**,**G** and **J**), Tannat (**B**,**E**,**H** and **K**) and Sagrantino (**C**,**F**,**I** and **L**). (**A**–**C**) Tryptophol set: TOL/TOL-SO_3_H; (**D**–**F**) Indole lactic acid set: ILA/ILA-SO_3_H; (**G**–**I**) Monomeric flavanol set: ECAT/ECAT-SO_3_H; (**J**–**L**) Procyanidin type B2 set: PROC-B2/PROC-B-SO_3_H.

**Figure 6:**
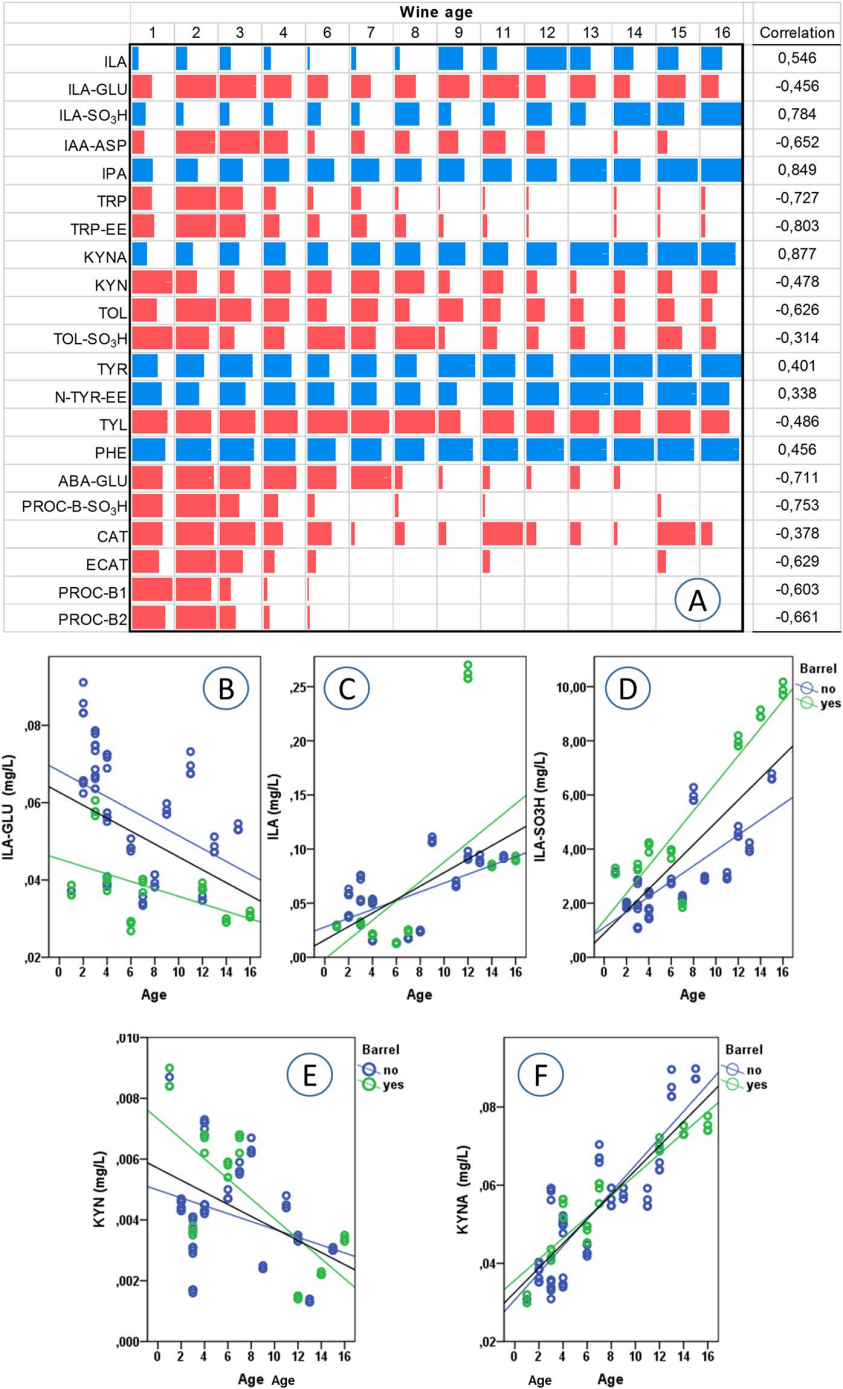
(**A**) Data bar graphs with the trend for the principal metabolites in Verdicchio wine in relation to wine age, and the correlation of each metabolite with wine age. Red bars indicate metabolites having a negative correlation with age, while blue bars have a positive correlation. Longer bars represent higher values. (**B**–**F**) B-F show the behaviour of specific metabolites in Verdicchio wines in relation to wine age and the use of wood barrels during winemaking. Full statistics are given in Supplementary Tables S6 and S15.

**Figure 7:**
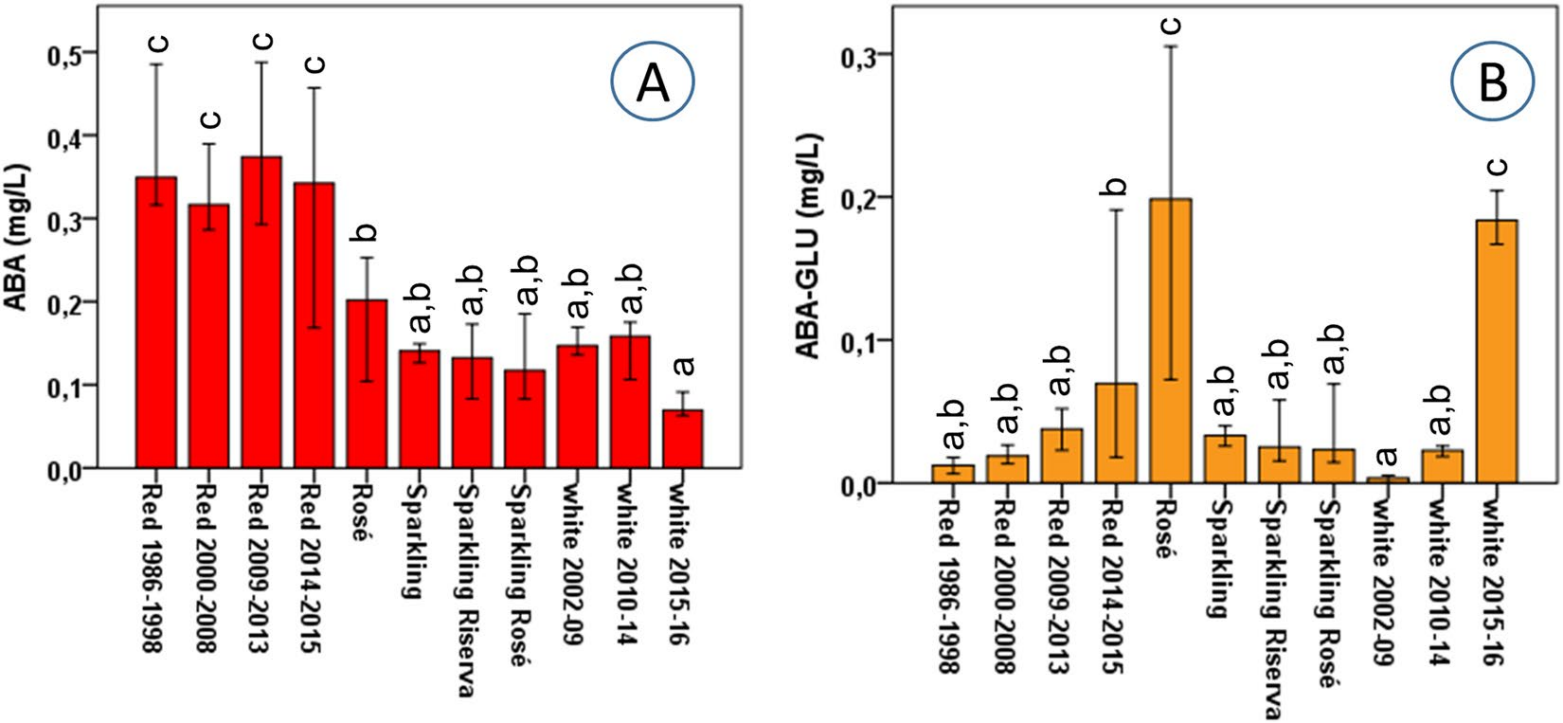
ABA (**A**) and ABA-GLU (**B**) mean concentration for the various groups of wine. The error bars represent a 95% confidence interval (Supplementary Table S3). Means not sharing a letter are significantly different (Supplementary Table S4).

Figures 2–4 present an overall view of the results reported in detail in Supplementary Table S3 and Figure S13. The 195 wines were separated based on their age/vintage and the type of winemaking (red, rosé, sparkling and white), in order to obtain some initial general considerations regarding the major reactions in Fig. 1, by grouping together very different commercial samples. Figure 2 also shows the results of statistical analysis, demonstrating the statistical significance of the various wine groups (full statistical analysis can be found in Supplementary Table S4). On the other hand, Figs 5 and 6 focus on specific varieties/wines. Figure 5 demonstrates the relative ratio between the major sulfonation reactions in Fig. 1 for three red wines, namely Tannat, Sagrantino and Amarone (the first two came from different wineries, while all the Amarone wines were from the same winery). Figure 6 is dedicated to the white wine Verdicchio, including four different product lines from the same winery, and covering wines aged for up to 16 years (2001–2016 vintages). Once again, the upper part of the figure (Fig. 6A) gives an overall picture of the chemical changes using a bar graph (which is similar to a heat-map graph), while the lower part of the figure (Fig. 6B–F) focuses on ILA derivatives and KYN/KYNA, which concentrations had good correlation with the wine age. Finally, Fig. 7 shows the behaviour of ABA (abscisic acid) and its glucoside ABA-GLU in general (Fig. 7A,B), and specifically for the four wines (Fig. 7C–F).

This project tried to provide an updated quantitative snapshot of the metabolic products of three essential amino acids (tryptophan, phenylalanine and tyrosine) present in wines and to elucidate their interaction with SO_2_, which is the gold standard additive for wine quality protection^1,7^. Moreover, it highlights the important presence of sulfonated flavanols in wine. Figure 1 summarises the various biological and chemical reactions delivering the majority of metabolites included in this work. The left-hand panel in the figure is dedicated to plant metabolism^1,15,33^, with which, starting from the shikimate pathway, the plant produces: a) three essential amino acids; b) the most important plant growth regulator IAA, its precursor ILA and their conjugates IAA-ASP and ILA-GLU; and c) grape tannins that have an enormous influence on wine quality and nutritional value^1,7,12^. The middle panel in Fig. 1 depicts the products of tryptophan catabolism by yeast during fermentation^20,23^, although these enzymatic reactions can also occur in grapes or take place in wine through chemical transformation. Finally, the right-hand panel shows novel or recently discovered chemical reactions occurring in wines due to the presence of SO_2_^9,14^. To our knowledge, this is the first time that ILA-SO_3_H, IAA-SO_3_H, KYN, KYNA, N-TYR-EE and ABA-GLU have been detected in wine, while in addition to this, the compounds TOL- SO_3_H, ECAT-SO_3_H and PROC-B-SO_3_H were also quantified in wine for the first time.

At wine pH (typically 3.0–3.7), SO_2_ is generally present in the form of its bisulfite (HSO_3_^−^), which reacts with several electrophilic wine components as a nucleophile. The oenological textbooks classify SO_2_ binders as weak and strong, with acetaldehyde being considered as the main binder due to its concentration in wine and its dissociation constant. Other well-characterised SO_2_-binders in wine are pyruvic acid, 2-ketoglutaric acid, glyceraldehyde, acetoin, glucose, fructose, galacturonic acid and anthocyanins^1,7^. In the last few years, some indolic^9^ and phenolic^10,14,32,34,35^ wine components have been added to this list as SO_2_-binders.

The first message of this study is that the reactivity/behaviour of SO_2_ changes according to the metabolic environment. Wine is a unique food matrix, in which grapes, yeasts, bacteria, oxygen, chemistry, wood and humans collaborate to deliver one of the richest matrices in terms of the number of metabolites. The few thousands of known^1,7,33,36^ metabolites are just the tip of the iceberg, because many detectable metabolites are unknown and many more are undetectable with current analytical technology. By participating in the wine metabolome, all these metabolites generate a chemical equilibrium. Therefore, there are numerous chemical changes every time an external or internal factor “disturbs” the equilibrium, and these can vary from one wine to another. If we define wine quality as the extent to which all the established requirements relating to the characteristics of the food are met, this includes both external factors such as appearance, texture, and flavour, and internal factors (chemical, physical and microbial). Since wine can be divided up into its individual components (chemical fingerprint/metabolome), its quality can be explained by measuring the individual components. Thus, the modification of each chemical constituent influences wine quality to some extent, and the modification of compounds with a high impact on a single quality factor (e.g. tannins in wine texture) will have a greater impact on wine quality. Wine quality terms have been used in this chemical perspective.

In this project we decided to go a step further compared to our former works^9,10,14^ and further validate our previous hypothesis with a deductive experimental design, including a wide variability of real/commercial samples and increasing the number of sulfonated compounds.

The sample set (Supplementary Table S3) contained 195 different commercial red, rosé, white and sparkling wines from 19 grape varieties produced between 1986 and 2016, creating a unique sample set able to deliver conclusions applicable to a wide range of wines on the global market. The distribution shown with error bars in the graphs in Fig. 2 was the result of extensive sampling and wide biological variability. Thus, each error bar includes biological variability due to vintage, biological variability due to variety, biological variability due to the winemaking process and the minor instrumental variability.

The general outcome of this work, which will be discussed subsequently in detail, demonstrated that on one hand the sulfonation of metabolites with an indole scaffold dominated in white wines, and on the other hand in red wines, which contain large amounts of red pigment anthocyanins, there was a lower concentration of sulfonated indoles, and SO_2_ reacted preferably with epicatechin and procyanidin B2, at a relatively slow rate. Therefore, the second message of this study was that although we are talking about the same food matrix - wine - the behaviour of the red and white wine metabolomes is different. This is why the reactions occurring in model wine solution (hydro-alcoholic solution with tartaric acid and pH around 3) often fail to explain the phenomena occurring in wine.

As expected, red wines had a much higher concentration in flavanols (CAT, ECAT, PROC-B1 and PROC-B2), due to the winemaking process, and their concentration decreased with the age of the wine (Figs 2 and 4 – Supplementary Table S4)^1,7^. Therefore, it was predictable that red wines should also have a higher concentration of their sulfonated products. In graphs M and O in Fig. 2, we can note that the concentration of ECAT-SO_3_H can reach values above 10 mg/L in red wines aged for over 10 years, and the average concentration of the dimer PROC-B-SO_3_H was 3 mg/L. The absolute concentration of ECAT, PROC-B2 and PROC-B-SO_3_H decreased with wine age for all wines, while ECAT-SO_3_H showed a tendency to increase with age in Amarone and Tannat (Figs 2L–O, 4 and 6 - Supplementary Figs S13–15 and Tables S4 and 5). However, from the sulfonated/unsulfonated ratios of ECAT and PROC-B2, it is clear that as age increased the balance progressed towards sulfonated products, both when considering all the wines together (Fig. 3E,F) or each wine separately (Fig. 5G–I - Supplementary Fig. S16). In white wines, the PROC-B-SO_3_H/ PROC-B2 ratio tends to favour faster the sulfonation in relation to the ECAT-SO_3_H/ ECAT equilibrium (Fig. 3E,F). If we consider only white Verdicchio wines, sulfonated PROC-B-SO_3_H covered about 40% in young wines (1, 2 years old), but this % increased with age and arrived at 100% for 8-year-old Verdicchio (Supplementary Fig. S16). Sparkling wines behaved more similarly to white wines, although no ECAT-SO_3_H and a very low concentration of ECAT were detected in the former (Figs 2–4 and Supplementary Table S4). In red wines, the ECAT-SO_3_H/ ECAT ratio already appeared to favour the sulfonated product after a few years of ageing, while sulfonated dimers took advantage of the PROC-B-SO_3_H/ PROC-B ratio after a decade of ageing (Fig. 3E,F). This general behaviour in red wines was also confirmed when each individual wine type/variety was considered separately, and especially if we focus on Amarone and Tannat wines, where the age range is wider (Fig. 5G–L). In Amarone and Verdicchio wines, where sampling included several vintages and all the samples of each wine came from the same winery, statistical analysis indicated a strong correlation between age and sulfonated/unsulfonated monomeric/dimeric flavanols (Fig. 6A - Supplementary Table S5).

The sulfonation of monomeric and dimeric flavanols is a reaction favoured by temperature, and wines of the same age stored in different conditions will have significantly different concentrations. Specifically, red wines stored at higher temperature will contain more ECAT-SO_3_H and PROC-B-SO_3_H, and their chemical age will appear older^14^. In the light of the stereochemical requirements discussed previously^10^, we suggest this may be a general reaction involving any monomeric, dimeric, oligomeric or polymeric flavanols on their terminal C4 position, provided that they have the “epi” configuration. Particularly, Mattivi *et al*.^10^ demonstrated that the slow reaction of a mixture of monomeric and polymeric flavanols with SO_2_ and temperature in the range 20–60 °C is favoured by temperature and takes place in the C ring, specifically at the C-4 position of the epicatechin flavanic structure. Two peaks, corresponding to the mono-sulfonated epicatechin and the main epicatechin-dimer sulfonated, were purified and subjected to NMR characterisation. As shown by the extensive NMR mono- and bi-dimensional experiments, only a β-substituent at C-4 would be able to impose a strong shielding γ-gauche effect on the C-2 of epicatechin. The main dimeric compound produced by the same reaction was found to be procyanidin B2 4β-sulfonate, with sulfonation on the terminal unit, thus retaining the same stereochemistry as in epicatechin 4β-sulfonate. This highlights the importance of sterical constraint of the flavanols cis-configuration for the reaction mechanism.

High quality red wines are rich in monomeric/oligomeric flavanols that have an astringent and bitter taste when young, but with ageing the monomeric/oligomeric flavanols participate in several reactions with other wine components (i.e. anthocyanins, acetaldehyde), undergoing polymerisation and finally changing the sensorial characteristics of the wine, to become less aggressive and smoother^1,7^. Alongside these reactions, we should now add sulfonated monomeric and dimeric flavanols, which are expected to modify reactivity, given the major changes in the polarity of terminal units, those most exposed to direct interaction with proteins. Considering both the concentration of sulfonated compounds and their tendency to increase with wine age, we could assume that sulfonated flavanols, a class of compounds so far neglected, could play a role in improving the sensorial quality of red wine with ageing. Such a hypothesis would be even more promising if we combined knowledge about how sulfonation modifies the biological activity of medicinal or toxic compounds^4–6^ with evidence of interaction between flavanols and salivary proteins to explain their contribution to mouthfeel^1,37,38^. In addition, Foo *et al*.^13^, demonstrated the cleavage of the dimeric flavanols interflavanic bond in the presence SO_2_, realising sulfonated and un-sulfonated monomers. This was also confirmed by the work of Mattivi *et al*.^10^. If such reaction occurs also in wine (which is very probable), the depolymerisation of the condensed tannins will further influence wine mouthfeel. Further work is needed to establish their contribution to wine taste and stability.

The second group of metabolites influenced by SO_2_ in wine (especially white wines) included the amino acid TRP and its catabolites/products (Fig. 1). In terms of TRP, sparkling wines had much lower amounts compared to other wines, probably due to second fermentation (Figs 2G and 4 – Supplementary Table S4)^1^. The concentration of TRP in both red and white wines decreased with age, and its ethyl ester TRP-EE showed similar behaviour (Figs 2G,H and 4 – Supplementary Tables S4 and Fig. S13). This trend/correlation with wine age was also confirmed when individual types of red or white wines were considered (Fig. 6 – Supplementary Table S5 and Figs S15 and S17).

Generally speaking, TOL-SO_3_H (Fig. 1) was quantified in higher amounts in young white and rosé wines, with a concentration above 3 mg/L (Figs 2B and 4 - Supplementary Table S4). The formation of TOL decreased with age in both red and white wines, with a high level of sulfonation in young white wines, indicating that TOL-SO_3_H may also give rise to further products similar to 2 AA (Figs 2A,B, 3A and 4). Verdicchio wines confirmed this general behaviour in white wines (Fig. 6A - Supplementary Table S5 and Fig S18). Similarly, red Amarone, Tannat and Sagrantino wines confirmed that with age the sulfonated/unsulfonated TOL ratio favoured TOL-SO_3_H (Figs 3A, 5A–C). It should be pointed out that when comparing the three syntheses described in the Methods section, TOL was more reactive than IAA and ILA. Under the same conditions, the sulfonation of tryptophol required less time (2 days) and less sodium bisulfite (50 mg) compared to ILA (6 days and 200 mg) and IAA (14 days and 300 mg).

The ILA and ILA-SO_3_H pairing showed similar behaviour to the TOL and TOL-SO_3_H pairing. In red wines both ILA and ILA-GLU decreased with age, while sulfonated ILA-SO_3_H reached a higher concentration in very old red wines (Fig. 2C,D - Supplementary Table S4 and Fig. S19). ILA-GLU is synthesised from the plant, but like other glucosides^10,14^, the glucosidic bond is hydrolysed later in wine, producing the aglycon ILA that later reacts with SO_2_ to deliver ILA-SO_3_H (Fig. 1). As regards young wines, reds had a much higher concentration of ILA and especially ILA-GLU, together with rosé, probably due to maceration with the skins during winemaking. White and especially sparkling wines instead had the lowest concentration, due to soft pressing and secondary fermentation in the bottle, which necessitates further nitrogen consumption (Fig. 2C - Supplementary Table S4 and Fig. S17G). The average concentration of the sulfonated product ILA-SO_3_H reached 10 mg/L (Fig. 2D). Figures 6A and D show the strong correlation (Supplementary Table S5) between Verdicchio wine age and ILA-SO_3_H, which is even stronger for wines of the same variety aged in wooden barrels, probably due to the micro-oxygenation process^9^.

Sulfonated IAA-SO_3_H was only detected in white and sparkling wines, at a concentration between 0.1 and 0.2 mg/L, while its parent IAA was detectable only in red wines, showing that this reaction is the preferred route in white and sparkling wines, but not in reds (Fig. 2E,F and Supplementary Table S4).

Considering the quantitative ratio between the free form of the three indoles and their sulfonated derivatives, it is clear from Fig. 3A–C that in white and sparkling wines the equilibrium favours the accumulation of sulfonated derivatives, compared to red and rosé wines. We can conclude that the recently discovered TOL-SO_3_H, ILA-SO_3_H and IAA-SO_3_H are all present in white wines at a much higher concentration than their unsulfonated precursors.

Hoeniche *et al*.^39^ proved that the sulfonation of indoles in model wine solution (i.e. IAA) could be responsible for their degradation and the formation of aromatic aminobenzenes such as 2AA, which could be responsible for some of the heavy aromatic characteristics of aged wines. So, the question arising is whether all the sulfonated indoles in Fig. 1 could also be precursors of other heavy aromatic compounds that take part in wine sensorial characteristics. Taking into account the fact that TOL-SO_3_H is not accumulated in white wines, like IAA-SO_3_H and ILA-SO_3_H, and the high concentration of TOL compared to IAA, along with their structural similarity, it could be surmised that TOL sulfonation could also deliver 2AA (or other similar aromatic compounds).

Moreover, our findings based on the quantification of sulfonated indoles could be the key to understanding the unusual behaviour of some wines in terms of consuming more SO_2_ than expected^40^. Lately, various research groups have focused on understanding SO_2_ consumption in wine, because the chemometric models established in solution have failed to explain what happens in wine^40–43^. Our results could lead to future applications in the winemaking process. The recommendations about the minimum necessary SO_2_ doses take in account very general characteristic of the wine, such as wine style (red, white, sparkling, etc) and pH. Therefore, often expert/veteran oenologists use SO_2_ based in empirical knowledge. The novel information on the presence of several compounds capable of reacting with SO_2_ in wine could be helpful in the wine industry in relation to targeted, finely tuned SO_2_ addition, building differentiated protocols for different wines with the final aim of decreasing SO_2_/sulfites in wine. At this point, we should underline that IAA and ILA derivatives are mainly inherited in wine from grapes, so are highly dependent on climate and the grape cultivar, while TOL and TRP derivatives are principally produced during winemaking, so are dependent on yeasts and fermentation conditions (Fig. 1)^1,20,44,45^.

This tendency for sulfonated compounds to increase with ageing, both at qualitative and quantitative level, could be the key to explaining the molecular oeno-diagenesis process during ageing, in which CHONS chemical spaces exhibited higher diversity in older wines, whereas there appeared to be fewer nitrogen-containing compounds (CHON chemical space)^35,46^.

KYNA is currently considered to be a product of yeast fermentation during winemaking^47^, but according to the results of this experiment, in which the concentration of KYNA increased with age in both red and white wine, we should now assume that chemical reactions occurring during wine ageing are able to produce KYNA. Indeed, Fig. 6A and F show a very strong correlation between KYNA concentration and Verdicchio wine age (Supplementary Table S5). Red and rosé wines had higher amounts of KYNA compared to white and sparkling wines, and all had concentrations similar to other foods^48^.

The last part of this manuscript is dedicated to the plant hormone ABA and its glucoside ABA-GLU (Figs 1 and 7). Like many important aromatic wine compounds (i.e. norisoprenoids), ABA is an enzymatic product of carotenoids and can be found in both free and glucosidic form in grapes^33,49^. Here we provide a major survey for the first time, in terms of the number, age and type of wines in relation to the ABA-GLU concentration. In plants, ABA biosynthesis is also correlated with the accumulation of polyphenols, and especially red anthocyanins^33,49^, so it is normal to quantify a higher amount of both ABA and ABA-GLU in red and rosé wines (Fig. 7 - Supplementary Table S4). Some interesting findings emerging from this study were that the ABA-GLU concentration was comparable in young red, rosé and white wines, and that the concentration of ABA is more stable during wine ageing in comparison to ABA-GLU, which decreased rapidly. The same behaviour was recorded when all or individual white or red wine varieties/types were considered (Fig. 6 – Supplementary Table S4 and 5 and Fig. 20). Information on ABA and ABA-GLU could provide good indications for understanding how other carotenoid breakdown products, such as aromatic norisoprenoids, behave during wine ageing.

In conclusion, this study opens new doors for better understanding the chemical changes occurring in wine during ageing and focuses specifically on the interaction of several wine metabolites with SO_2_. This knowledge paves the way for smarter use of this important chemical in winemaking. Nine metabolites (ILA-SO_3_H, IAA-SO_3_H, KYN, KYNA, N-TYR-EE, ABA-GLU, TOL-SO_3_H, ECAT-SO_3_H and PROC-B-SO_3_H) were quantified for the first time in wine, in a large sample set of 195 wines of various types. Six of these metabolites (ILA-SO_3_H, IAA-SO_3_H, KYN, KYNA, N-TYR-EE and ABA-GLU) were also identified for the first time in wine, while the synthesis of two compounds (ILA-SO_3_H and TOL-SO_3_H) was reported for the first time. It was clarified that the white wine metabolome, which lacks anthocyanins and has a low flavanol content, preferably interacts with SO_2_ through the sulfonation of indoles, while the red wine metabolome interacts with SO_2,_ producing among other things sulfonated monomeric and oligomeric flavanols. This latter reaction could be the key to understanding the desirable smoothing of aggressive young red wines with ageing. Tryptophan catabolite behaviour during ageing could help to avoid several wine faults and decrease sulfites/SO_2_. In general terms, this work demonstrates that the presence of SO_2_ in wine influences wine quality in more ways than is currently known, and that the high concentration of sulfonates in aged wines is strong evidence of this influence. SO_2_ chemistry/effects in wine should be re-evaluated.

## Methods

### Wine samples

The sample list included 195 commercial wines produced from 1986 until 2016, covering the most common styles of wine, and was made up of 93 white wines, including light-bodied and full-bodied white wines (35 Chardonnay, 32 Pinot gris, 24 Verdicchio and 2 others), 60 reds including light-bodied, medium-bodied and full-bodied red wines (18 Sagrantino, 13 Tannat, 12 Sangiovese, 11 Amarone and 6 others), 37 sparkling wines (including white and rosè sparkling wine), and 5 still rosé wines. Further information and details about the wines are reported in Supplementary Table S3.

### Organic synthesis

^1^H (400 MHz), ^13^C (100 MHz) and 2D-NMR spectra (HSQC and HMBC) were recorded at 300 K in CD_3_OD (99.9%) or in D_2_O (99.5%) or in CD_3_OD/D_2_O 2: 1 on a Bruker-Avance 400 MHz NMR spectrometer, using a 5 mm BBI probe equipped with pulsed-gradient field utility. The chemical shift scale (δ) was calibrated on the residual proton signal of deuterated acetone (δ_H_ 2.050 ppm and δ_C_ 29.80 ppm), tetradeuterated methanol (δ_H_ 3.310 ppm and δ_C_ 49.00 ppm) or deuterated water (δ_H_ 4.75 ppm). All the NMR spectra can be found as supplementary data (Figs S3–14).

For exact mass measurements the Synapt HDMS QTOF MS (Waters, Manchester, UK) was used and the parameters are described in Arapitsas *et al*.^14^.

#### *Tryptophol-2-sulfonate (TOL-SO*_3_*H) [*3*-(2-hydroxyethyl)-1H-indole-2-sulfonate]*

A TOL solution (25 mg in 2 mL ethanol), which corresponds to a molar ratio of 3.1:1, was poured slowly into a sodium bisulfite solution (50 mg in 5 mL H_2_O) at room temperature, while stirring. The reaction was monitored with LC-MS control of tryptophol consumption. After 2 days, the reaction solution was concentrated until dry by evaporation under reduced pressure at 30 °C, reconstituted in 2 mL of water, and the product was purified with an ENV + cartridge. Purification was achieved as follows: the cartridge was activated with 10 mL of methanol and conditioned with 20 mL of water, 2 mL of the sample was loaded, then the cartridge was washed with 5 mL of water and the sulfonated product was eluted with 20% methanol. This process produced 14 mg of TOL-SO_3_H.

^1^H-NMR (D_2_O, 300 K): 7.75 (dt, J = 8.1, 1.0 Hz, 1 H, H-6), 7.51 (dt, J = 8.3, 0.9 Hz, 1 H, H-9), 7.35 (ddd, J = 8.3, 7.0, 1.2 Hz, 1 H, H-8), 7.21 (ddd, J = 8.1, 7.0, 1.0 Hz, 1 H, H-7), 3.85 (t, J = 7.2 Hz, 2 H, 2H-11), 3.26 (t, J = 7.3 Hz, 2 H, 2H-10).

^13^C-NMR (D_2_O, 300 K): 136.06 (C-5), 135.18 (C-2), 128.62 (C-4), 125.75 (C-8), 121.56 (C-7), 121.50 (C-6), 113.68 (C-9), 113.44 (C-3), 63.41 (C-11), 28.05 (C-10).

HRMS (m/z): [M–H]^−^ calcd. for C_10_H_11_NO_4_S 240.0336, found 240.0337.

#### *Indole-lactic acid-2-sulfonate (ILA-SO*_3_*H) [3-(2-carboxy-2-hydroxyethyl)-1H-indole-2-sulfonate]*

An ILA solution (26 mg in 2 mL ethanol), which corresponds to a molar ratio of 12.9:1, was poured slowly into a sodium bisulfite solution (200 mg in 5 mL H_2_O) at room temperature, while stirring. The reaction was monitored with LC-MS control of indole lactic acid consumption. After 6 days, the reaction solution was concentrated until dry by evaporation under reduced pressure at 30 °C, reconstituted in 2 mL of water, and the product was purified with an ENV + cartridge. Purification was achieved as previously described for TOL-SO_3_H. This process produced 30 mg of ILA-SO_3_H.

^1^H-NMR (CD_3_OD, 300 K): 7.55 (brd, J = 8.3 Hz, 1 H, H-6), 7.38 (brd, J = 8.2 Hz, 1 H,H-9), 7.17 (t, J = 8.2 Hz, 1 H, H-8), 7.04 (t, J = 8.2 Hz, 1 H,H-7), 4.55 (dd, J = 5.8,7.8, 1 H, H-11), 3.51 (dd, J = 5.8, 14.2 Hz, H-10a), 3.45 (dd, J = 7.8,14.2, 1 H, H-10b).

^13^C-NMR (CD_3_OD, 300 K): 176.52 (C-12), 137.95 (C-2), 136.14 (C-5), 128.98 (C-4), 124.23 (C-8), 120.63 (C-7), 120.48 (C-6), 112.85 (C-3), 110.13 (C-9), 72.69 (C-11), 30.52 (C-10).

HRMS (m/z): [M–H]^−^ calcd for C_11_H_11_NO_6_S 284.0229, found 284.0221.

#### Indole-acetic acid-2-sulfonate (IAA-SO_3_H) [3-(carboxymethyl)-1H-indole-2-sulfonate

An IAA solution (27 mg in 2 mL ethanol), which corresponds to a molar ratio of 21.9:1, was poured slowly into a sodium bisulfite solution (300 mg in 5 mL H_2_O) at room temperature, while stirring. The reaction was monitored by LC-MS control of indole lactic acid consumption. After 14 days, the reaction solution was concentrated until dry by evaporation under reduced pressure at 30 °C, reconstituted in 2 mL of water, and the product was purified with an ENV + cartridge. Purification was achieved as follows as previously described for TOL-SO_3_H. This process produced 12 mg of IAA-SO_3_H.

^1^H NMR (CD_3_OD/D_2_O 2/1, 300 K): 7.62 (d, J = 8.0 Hz, 1 H, H-6), 7.42 (d, J = 8.2 Hz, 1 H,H-9), 7.23 (t, J = 8.2 Hz, 1 H, H-8), 7.08 (t, J = 8.2 Hz, 1 H,H-7), 3.93 (s, 1 H, 2H-10)

^13^C-NMR (CD_3_OD/D_2_O 2/1, 300 K): 180.61 (C-11), 135.98 (C-5), 135.57 (C-2), 128.83 (C-4), 124.56 (C-8), 121.32 (C-6), 120.53 (C-7), 112.73 (C-9), 112.00 (C-3), 33.97 (C-10).

HRMS (m/z): [M–H]^−^ calcd for C_10_H_9_NO_5_S 254.0129, found 254.0136.

The purity of all three metabolites was determined using NMR and was higher than 95%.

### UHPLC-MS/MS analysis

UHPLC-MS/MS analysis was performed on a Waters Acquity UPLC system (Milford, MA). The method was based on the combination of two previous methods^26,29^, and small modifications were made in order to add new metabolites. Separation of phenolic compounds was achieved on a Waters Acquity HSS T3 column 1.8 μm, 150 mm × 2.1 mm (Milford, MA, USA), kept at 40 °C. Mobile phase A was water containing 0.1% formic acid; mobile phase B was acetonitrile containing 0.1% formic acid. The flow was 0.4 mL/min, and the gradient profile was 0 min, 5% B; from 0 to 2 min, linear gradient to 20% B; from 2 to 3 min, isocratic 20% B; from 3 to 3.3 min, linear gradient to 28% B; from 3.3 to 8 min, linear gradient to 42% B; from 8 to 10 linear gradient to 100% B; from 10 to 12 min, wash at 100% B; from 12 to 12.1 min, back to the initial conditions of 5% B; finally the column was equilibrated for 2.5 min until the next injection. The injection volume of both the standard solutions and the samples was 10 μL. Samples were kept at 6 °C during the analysis.

Mass Spectrometry. Mass spectrometry detection was performed on a Waters Xevo TQMS (Milford, MA, USA) instrument equipped with an electrospray (ESI) source. Capillary voltage was 3.5 kV in positive mode and −2.7 kV in negative mode; the source was kept at 150 °C; desolvation temperature was 500 °C; cone gas flow, 50 L/h; and desolvation gas flow, 1000 L/h. Unit resolution was applied to each quadrupole. Flow injections of each individual metabolite were used to optimise the MRM conditions. For the majority of metabolites, this was done automatically with Waters Intellistart software, whereas for some compounds the optimal cone voltages and collision energies were identified during collision-induced dissociation (CID) experiments and manually set.

The samples were divided into three batches and analysed in two periods (February and May 2017). The first batch included QC_W_, white and sparkling wines; the second batch included QC_R_ and some of the red wines; and the third batch QC_R_ and the remaining red wines. The samples were randomised for each batch sequence and the QC sample was injected every ten real samples^30^. Data processing was done using Waters TargetLynx tools of MassLynx 4.1 software. Method validation was performed by studying the linear dynamic range, precision of the analysis, and limit of quantification (LOQ) for the standard compounds.

Calibration curves were constructed for each standard at eleven concentration levels, in a concentration range spanning >5 orders of magnitude, and by using the mobile phases (95% A and 5% B) for the dilutions. The LOQ for each compound was evaluated as the concentration at which the quantifier transition presented a signal-to-noise (S/N) ratio of >10. The matrix effect was determined with a recovery test. Briefly, the pooled QC_W_ and QC_R_ samples were spiked with two levels of each analyte and then measured with the same protocol as the real samples. QC injections were used to validate instrumental variability during the period of the analysis.

Wines were uncorked under nitrogen atmosphere and an aliquot was transferred into a 15 mL amber vial (filled to capacity)^9,14,36,50^. Then, again under nitrogen atmosphere, one quality control (QC_W_) pooled sample was prepared using 0.5 mL of each white wine sample and a second quality control (QC_R_) pooled sample was prepared using 0.5 mL of each red wine sample. Then 20 μL of the internal standard (10 mg 3-nitrotyrosine in 10 mL of MeOH) was added to 2 mL of each wine and filtered with 0.2 μm PTFE filters into a 2 mL amber vial (MS certified) prior to LC/MS analysis. The same procedure was followed for the blank, but instead of wine, 2 mL of Milli-Q water was used. Each sample was analysed in triplicate. Sample preparation and analysis were performed in randomised order as described in Ehrhardt *et al*.^30^.

Data processing was carried out using Waters MassLynx version 4.1 and TargetLynx software (Milford, MA). One-way ANOVA with post-hoc Tukey’s HSD statistical analysis and Pearson’s correlation analysis was performed using SPSS V19 (IBM Statistics).

### Data availability

The authors confirm that all relevant data have been included in the paper and/or its supplementary information files, while all data of minor relevance are available from the corresponding author following a reasonable request.

## Electronic supplementary material

Supplementary_Data Tables S1-5 and Figures S1-20

Dataset 1

Dataset 2
