# Supplementary material for: The impact of SO2 on wine flavanols and indoles in relation to wine style and age
Source: Sci Rep. 2018 Jan 16;8:858. doi: 10.1038/s41598-018-19185-5 (PMC5770432; doi:10.1038/s41598-018-19185-5)
Supplement: Supplementary file 1 — Supplementary_Data Tables S1-5 and Figures S1-20 [file 41598_2018_19185_MOESM1_ESM.pdf]

The impact of SO_2 on wine flavanols and indoles in relation to wine style and age

Panagiotis Arapitsas[1,*], Graziano Guella[2,3] & Fulvio Mattivi[1,2]

[1]Department of Food Quality and Nutrition, Research and Innovation Centre, Fondazione Edmund Mach (FEM), San Michele all'Adige, Italy

[2]Center Agriculture Food Environment, University of Trento, San Michele all'Adige, Italy

[3]Bioorganic Chemistry Laboratory, Department of Physics, University of Trento, Trento, Italy

*panagiotis.arapitsas@fmach.it, panagiotis.arapitsas@gmail.com

Supplementary Data

Abbreviations	
2AAP	2-aminoacetophenon
ABA	abscisic acid
ABA-GLU	glucoside of abscisic acid
CAT	catechin
CID	collision-induced dissociation
CV	coefficient of variation
ECAT	epicatechin
HDMS	high definition mass spectrometry
HMBC	heteronuclear multiple-bond correlation spectroscopy
HSQC	heteronuclear single quantum coherence spectroscopy
IAA	indole 3-acetic acid
IAA-ASP	indole 3-acetic acid conjugate with aspartic acid
ICA	indole 3-carboxaldehyde
ILA	indole 3-lactic acid
ILA-GLU	indole 3-lactic acid glucoside
IPA	indole 3-pyruvic acid
KYN	kynurenine
KYNA	kynurenic acid
LC-MS	liquid chromatography - mass spectrometry
LOQ	limit of quantification
MEL	melatonine
MRM	Multiple Reaction Monitoring
MS	mass spectrometry
NMR	nuclear magnetic resonance
N-SER	N-acetyl-serotonine
N-TRP-EE	N-acetyl-tryptophan-ethyl ester
N-TYR-EE	N-acetyl-tyrosine-ethyl ester
PHE	phenylalanine
PROC-B1	procyanidin B1
PROC-B2	procyanidin B2
QC	quality control
QCR	red wine quality control
QCS	sparkling wine quality control
QCW	white wine quality control
QTOF	quadrupole time-of-flight
SER	serotonine
TOL	tryptophol
TRP	tryptophan
TRP-EE	tryptophan-ethyl ester
TYL	tyrosol
TYR	tyrosine
TYR-EE	tyrosine-ethyl ester
UHPLC	ultra high pressure liquid chromatography

**Supplementary Table S1.** LC-MS parameters and quantification indices									
**Metabolite**[a]	**rt (min)**	**ESI mode**	**Cone Voltage**	**MRM transitions**[d]	**R^2**	**LOQ**[e] **(µg/L)**	**Order of linearity**	**Supplier**	**matrix effect (%)**
ILA	4,62	+	18	206,1 → 118,0 (20)<br>206,1 → 160,1 (10)	0,9938	0,08	4	Sigma-Aldrich	-11,8
ILA-GLU[b]	3,6	-	15	366,2 → 186,2 (20)<br>366,2 → 204,1 (20)	-	-	-	-	-
ILA-SO_3H	2,99	-	24	284,1 → 222,1 (18)<br>284,1 → 142,0 (26)	0,9597	312,29	3	Synthesis[c]	19,3
IAA	5,32	+	18	176,1 → 103,0 (28)<br>176,1 → 130,0 (12)	0,9852	8,89	5	Sigma-Aldrich	-6,3
IAA-ASP	3,92	+	14	291,2 → 130,1 (22)<br>291,2 → 134,0 (12)	0,9963	0,19	6	Olchemim	-1,5
IAA-SO_3H	3,25	-	28	254,1 → 130,1 (24)<br>254,1 → 210,1 (28)	0,9834	108,89	4	Synthesis[c]	12,8
IPA	4,39	+	16	204,1 → 130,0 (22)<br>204,1 → 158,0 (12)	0,9871	62,60	4	Sigma-Aldrich	9,3
ICA	5,04	+	22	146,1 → 118,0 (14)<br>146,1 → 91,0 (24)	0,9967	6,09	3	Sigma-Aldrich	-38,5
2AAP	6,02	+	20	136,1 → 118,0 (20)<br>136,1 → 43,0 (16)	0,9994	0,02	5	Sigma-Aldrich	-13,5
TRP	2,58	+	12	205,1 → 146,0 (18)<br>205,1 → 118,0 (26)	0,9901	2,20	4	Sigma-Aldrich	7,3
N-TRP-EE	6,95	+	18	275,2 → 159,1 (22)<br>275,2 → 201,1 (12)	0,9786	1,50	3	Sigma-Aldrich	-5,5
TRP-EE	4,45	+	14	233,1 → 174,1 (14)<br>233,1 → 159,1 (20)	0,9917	38,90	4	Sigma-Aldrich	8,9
MEL	5,13	+	16	233,2 → 174,1 (14)<br>233,2 → 159,0 (28)	0,9947	0,65	5	Sigma-Aldrich	20,8
SER	1,82	+	12	177,1 → 115,1 (28)<br>177,1 → 105,0 (26)	0,9931	1,40	5	Sigma-Aldrich	13,7
N-SER	3,17	+	16	219,1 → 115,0 (34)<br>219,1 → 160,0 (16)	0,9862	2,20	5	Sigma-Aldrich	-17,8
KYNA	2,75	+	22	190,1 → 144,0 (14)<br>190,1 → 116,0 (32)	0,9913	0,80	5	Sigma-Aldrich	8,0
KYN	2,05	+	14	209,1 →146,1 (16)<br>209,1 → 136,0 (12)	0,9784	0,40	5	Sigma-Aldrich	41,3
TOL	5,4	+	14	162,2 →144,0 (20)<br>162,2 → 127,1 (22)	0,9868	1,50	4	Sigma-Aldrich	6,3
TOL-SO_3H	3,28	-	32	240,1 → 160,1 (22)<br>240,1 → 130,1 (28)	0,9663	18,62	4	Synthesis[c]	6,4
TYR	1,44	+	18	182,2 → 136,1 (16)<br>182,2 → 91,0 (26)	0,9923	11,20	4	Sigma-Aldrich	2,2
TYR-EE	2,68	+	18	210,2 → 136,1 (14)<br>210,2 → 91,0 (26)	0,9733	2,50	3	Sigma-Aldrich	3,0
N-TYR-EE	4,69		14	252,2 → 136,0 (22)<br>252,2 → 178,1 (12)	0,9901	1,50	5	Sigma-Aldrich	16,8
TYL	3,07	+	24	121,1 → 103,0 (18)<br>121,1 → 93,0 (12)	0,9542	14,30	4	Sigma-Aldrich	31,0
PHE	2,05	+	16	166,2 → 120,1 (12)<br>166,2 → 103,0 (24)	0,966	1,70	4	Sigma-Aldrich	-12,3
ABA	6,07	+	8	265,3 → 229,2 (10)<br>265,3→ 201,2 (14)	0,9845	2,00	4	Sigma-Aldrich	5,5
ABA-GLU	4,5	-	18	425,2 →263,1 (10)<br>425,2 → 153,0 (20)	0,9842	2,40	4	Olchemim	-32,2
CAT	2,88	-	32	289,0 → 203,0 (20)<br>289,0 → 123,0 (33)	0,964	40,70	4	Sigma-Aldrich	13,1
ECAT	3,24	-	32	289,0 → 203,0 (20)<br>289,0 → 123,0 (33)	0,9211	37,50	4	Sigma-Aldrich	17,8
PROC-B1	2,54	-	32	577,1 → 425,0 (26)<br>577,1 → 289,0 (16)	0,9899	64,60	4	Extrasynthese	3,2
PROC-B2	2,97	-	32	577,1 → 425,0 (26)<br>577,1 → 289,0 (16)	0,9882	25,90	4	Extrasynthese	27,0
ECAT-SO_3H	1,88	-	30	369,1 → 289,0 (25)<br>369,1 → 203,0 (30)	0,9364	44,08	3	Synthesis[c]	23,5
PROC-B-SO_3H	2,13	-	30	657,1 → 369,1 (30)<br>657,1 → 289,0 (30)	0,9801	48,80	4	Synthesis[c]	5,2

[a]ILA: indole 3-lactic acid; GLU: glucose; IAA: indole 3-acetic acid; IPA: indole 3-pyruvic acid; ICA: indole 3-carbocaldehyde; TRP: tryptophan; TRP-EE: tryptophan ethyl ester; N-TRP-EE: N-acetyl-tryptophan ethyl ester; N-SER: N-acetyl serotonin; KYNA: kynurenic acid; KYN: kynurenine; TOL: tryptophol; TYR: tyrosine; TYR-EE: tyrosine ethyl ester; N-TYR-EE: N-acetyl-tyrosine ethyl ester; TYL: tyrosol; CAT: catechin; ECAT: epicatechin: PRO-B: procyanidin

[b]ILA-GLU was quantified as ILA. [c]Product of organic synthesis in the laboratory. [d]The first line is the MRM quantifier, the second line the MRM qualifier and in parenthesis the collision voltage of each MRM. [e]Limit of quantification

Supplementary Table S2. Analytes concentration and variation for the Quality Control (QC) sample injections. The results were based in 16 injections for the white wine QC (QC_W), 8 for the sparkling wine (QC_S) and 11 for the red wine (QC_R)

Metabolite[a]	QC_W (µg/L)	QC_W %CV	QC_S (µg/L)	QC_S %CV	QC_R (µg/L)	QC_R %CV
ILA	7,41	10,13	71,36	8,21	84,12	9,35
ILA-GLU	29,28	9,41	21,84	10,50	87,25	11,13
ILA-SO_3H	9078,53	6,95	11437,78	5,32	1942,19	0,59
IAA	n.d.		n.d.		n.d.	
IAA-ASP	2,24	32,91	3,09	31,53	4,96	36,53
IAA-SO_3H	287,65	6,16	154,21	7,33	n.d.	
IPA	n.d.		182,74	6,55	78,07	24,49
ICA	n.d.		7,65	3,03	7,50	4,78
2AAP	n.d.		0,13	12,89	n.d.	
TRP	401,50	2,30	20,51	3,50	242,45	9,80
N-TRP-EE	n.d.		n.d.		n.d.	
TRP-EE	21,45	4,82	1,14	38,83	14,03	35,23
MEL	n.d.		n.d.		n.d.	
SER	n.d.		n.d.		n.d.	
N-SER	23,66	12,02	19,65	12,14	14,64	15,35
KYNA	26,93	9,11	47,68	8,74	154,89	6,37
KYN	19,71	9,37	3,62	23,49	1,78	43,39
TOL	19,71	9,37	29,99	9,40	178,89	11,50
TOL-SO_3H	3814,04	7,18	1128,96	8,78	1018,37	6,32
TYR	2926,21	2,26	3774,60	6,02	1466,39	12,58
TYR-EE	153,65	3,26	211,86	7,88	175,40	13,33
N-TYR-EE	2,12	9,45	2,77	13,94	13,97	2,94
TYL	n.d.		1762,91	8,92	4245,22	15,59
PHE	813,58	0,98	922,22	2,40	790,76	3,21
ABA	153,21	5,23	128,04	4,14	238,66	4,84
ABA-GLU	167,98	8,18	32,86	16,60	27,97	31,87
CAT	340,66	8,40	70,62	28,05	1058,42	9,62
ECAT	778,47	7,50	37,15	14,09	1342,85	4,20
PROC-B1	2769,78	9,36	n.d.		12754,13	0,46
PROC-B2	574,07	9,14	25,36	28,82	3889,08	0,29
ECAT-SO_3H	n.d.		n.d.		12622,41	10,29
PROC-B2-SO_3H	n.d.		n.d.		4501,59	2,70

[a]ILA: indole 3-lactic acid; GLU: glucose; IAA: indole 3-acetic acid; IPA: indole 3-pyruvic acid; ICA: indole 3-carbocaldehyde; TRP: tryptophan; TRP-EE: tryptophan ethyl ester; N-TRP-EE: N-acetyl-tryptophan ethyl ester; N-SER: N-acetyl serotonin; KYNA: kynurenic acid; KYN: kynurenine; TOL: tryptophol; TYR: tyrosine; TYR-EE: tyrosine ethyl ester; N-TYR-EE: N-acetyl-tyrosine ethyl ester; TYL: tyrosol; CAT: catechin; ECAT: epicatechin: PRO-B: procyanidin type B. n.d.: not detected

Supplementary Table S5. Paterson correlation analysis between wine age and metabolites for Verdicchio and Amarone wines.

	Verdicchio white wine		Amarone red wine	
Metabolites	Correlation	p-value	Correlation	p-value
ILA	0,546	< 0,0001	-0,860	< 0,0001
ILA-GLU	-0,456	< 0,0001	-0,856	< 0,0001
ILA-SO3H	0,784	< 0,0001	0,595	0,0010
IAA-ASP	-0,652	< 0,0001	-	
IAA-SO3H	0,191	0,06271	-	
IPA	0,849	< 0,0001	-	
ICA	0,364	< 0,0001	-	
TRP	-0,727	< 0,0001	-0,742	< 0,0001
TRP-EE	-0,803	< 0,0001	-0,480	0,0104
N-TRP-EE	-0,092	0,37007	-0,893	< 0,0001
N-SER	-0,087	0,40138	-	
KYNA	0,877	< 0,0001	0,647	0,0003
KYN	-0,478	< 0,0001	0,389	0,0450
TOL	-0,626	< 0,0001	-0,870	< 0,0001
TOL-SO3H	-0,314	0,00186	0,735	< 0,0001
TYR	0,401	0,00005	0,579	0,0015
TYR-EE	0,029	0,78183	-0,760	< 0,0001
N-TYR-EE	0,338	0,00074	-0,257	0,1958
TYL	-0,486	< 0,0001	0,592	0,0011
PHE	0,456	< 0,0001	-0,601	0,0009
ABA	0,156	0,12892	-0,654	0,0002
ABA-GLU	-0,711	< 0,0001	-0,704	< 0,0001
ECAT-SO3H	0,166	0,10545	-0,088	0,6612
PROC-B-SO3H	-0,753	< 0,0001	-0,886	< 0,0001
CAT	-0,378	0,00015	-0,772	< 0,0001
ECAT	-0,629	< 0,0001	-0,830	< 0,0001
PROC-B1	-0,603	< 0,0001	-0,887	< 0,0001
PROC-B2	-0,661	< 0,0001	-0,866	< 0,0001

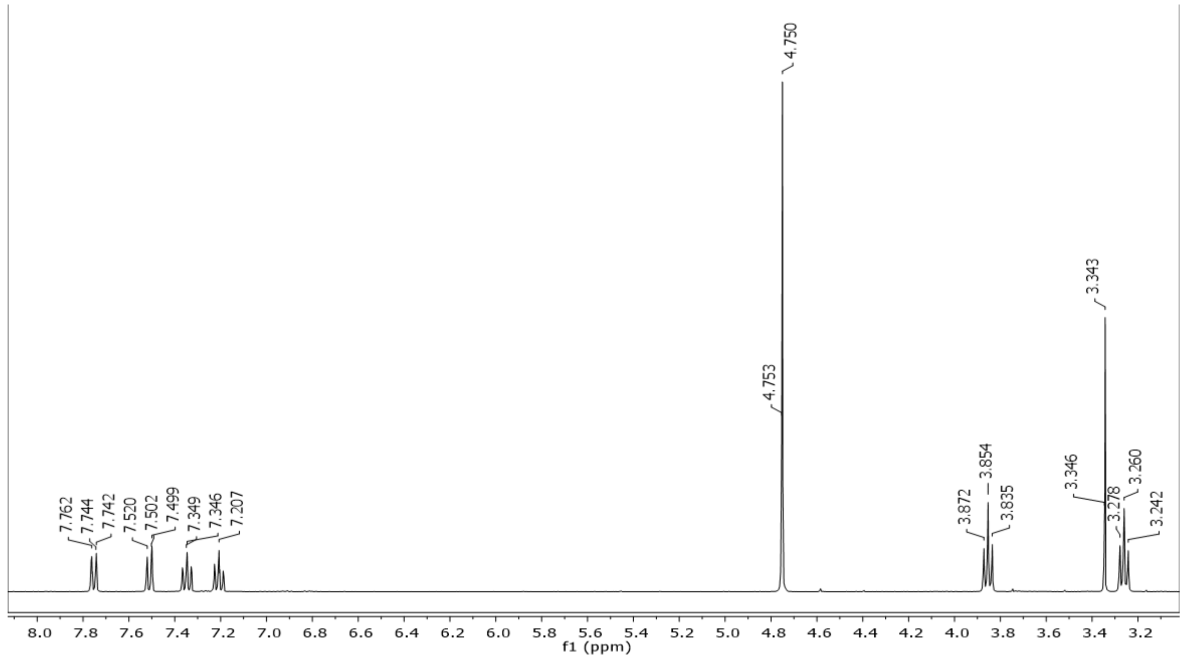

Figure S1. ^{1}H–NMR spectrum of TOL-SO_3H in D_2O

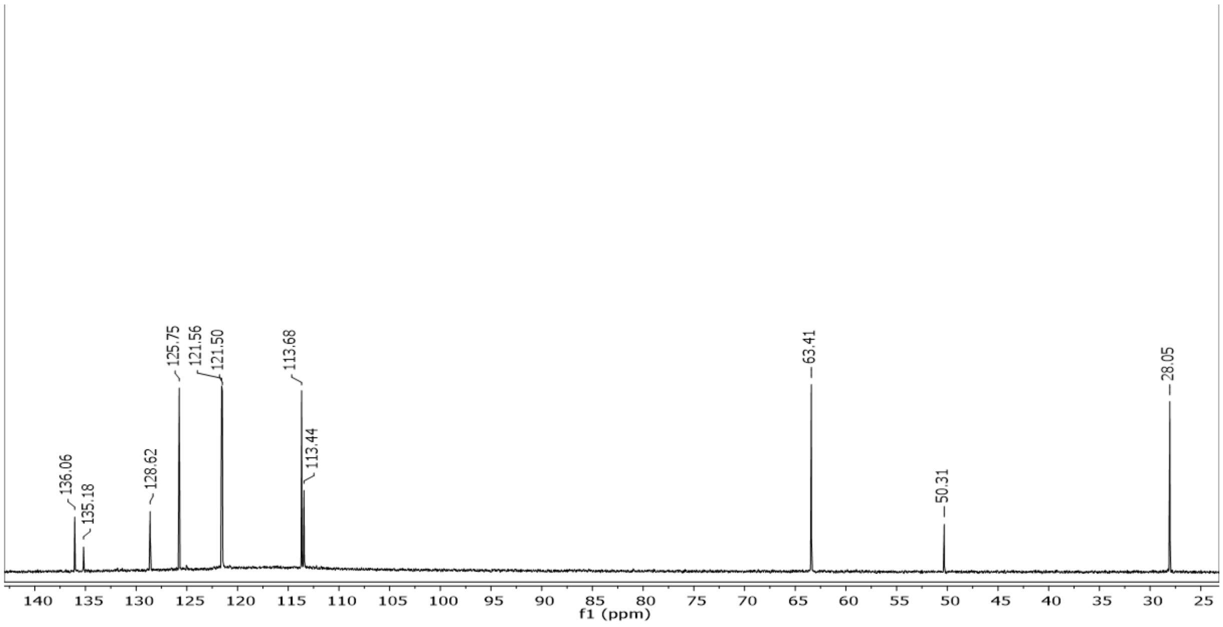

Figure S2. ^{13}C–NMR spectrum of TOL-SO_3H in D_2O

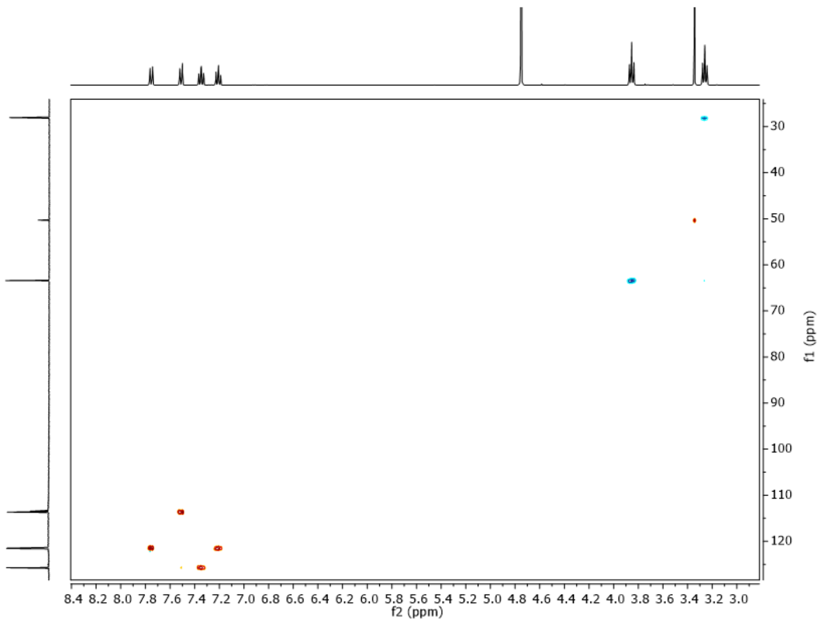

Figure S3. HSQC–2D-NMR spectrum of TOL-SO_3H in D_2O

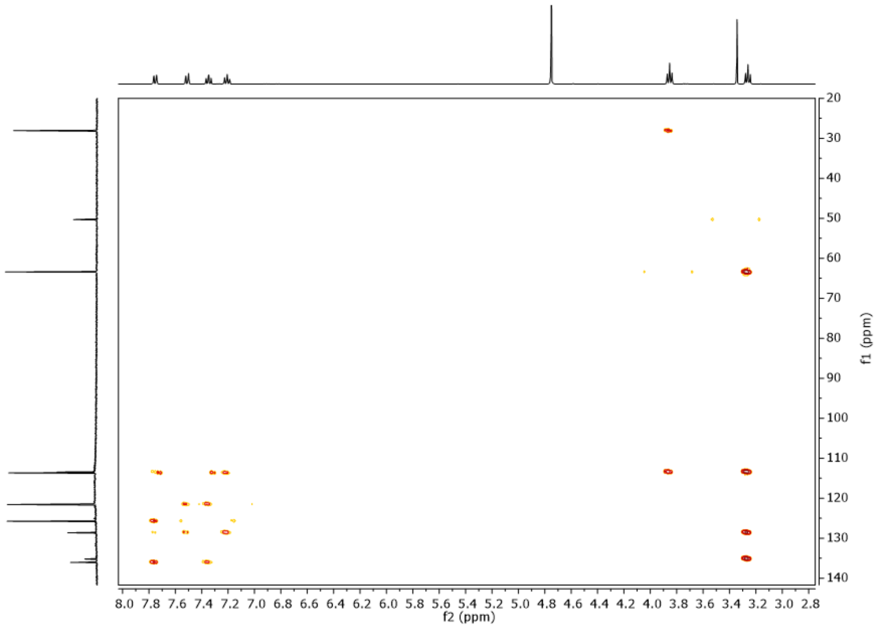

Figure S4. HMBC–2D-NMR spectrum of TOL-SO_3H in D_2O

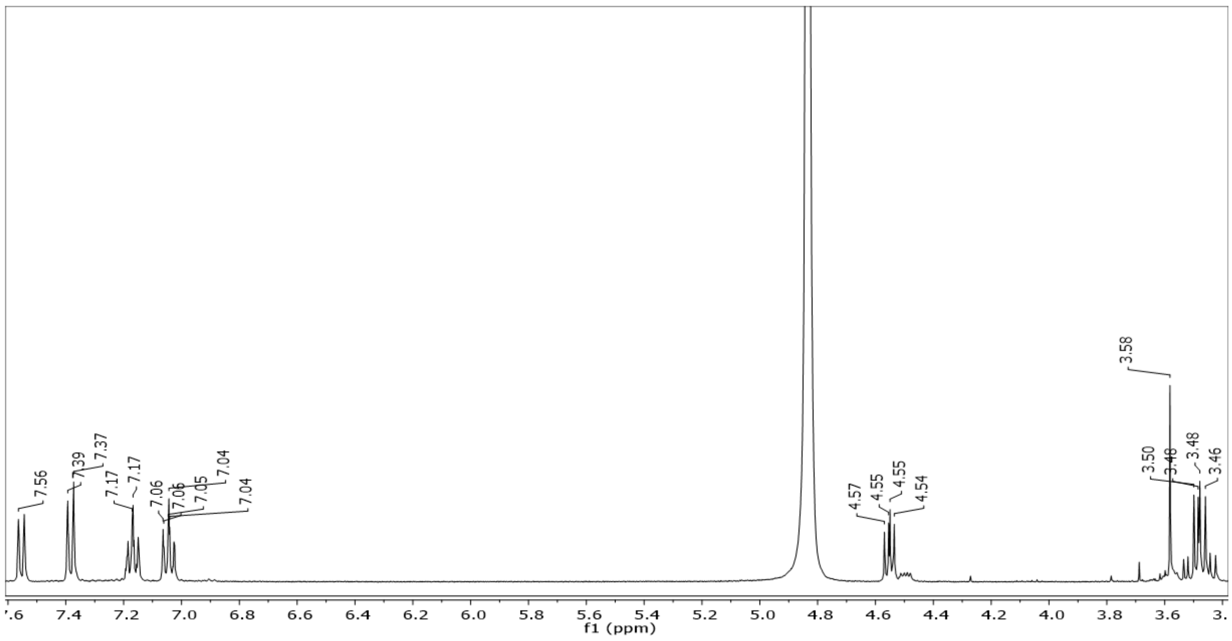

Figure S5. ^{1}H–NMR spectrum of ILA-SO_3H in CD_3OD

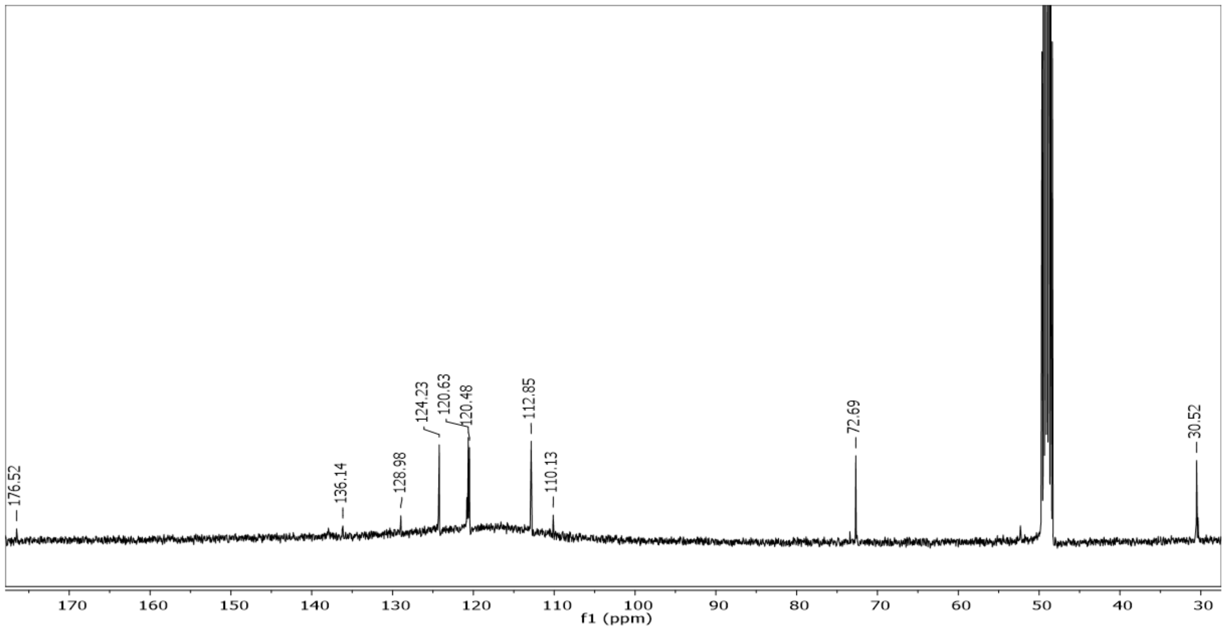

Figure S6. ^{13}C–NMR spectrum of ILA-SO_3H in CD_3OD

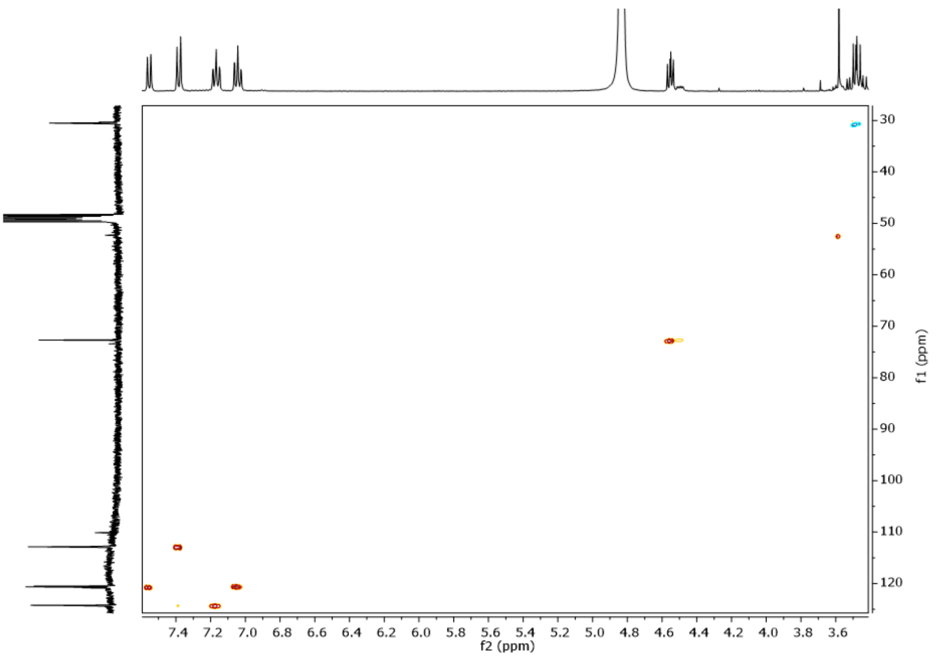

Figure S7. HSQC –2D-NMR spectrum of ILA-SO_3H in CD_3OD

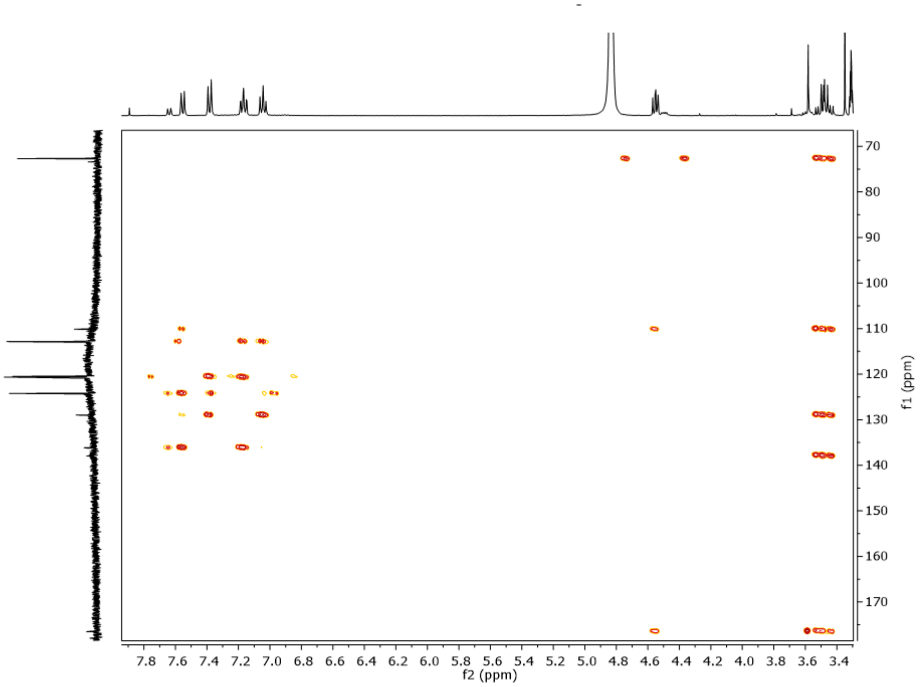

Figure S8. HMBC–2D-NMR spectrum of ILA-SO_3H in CD_3OD

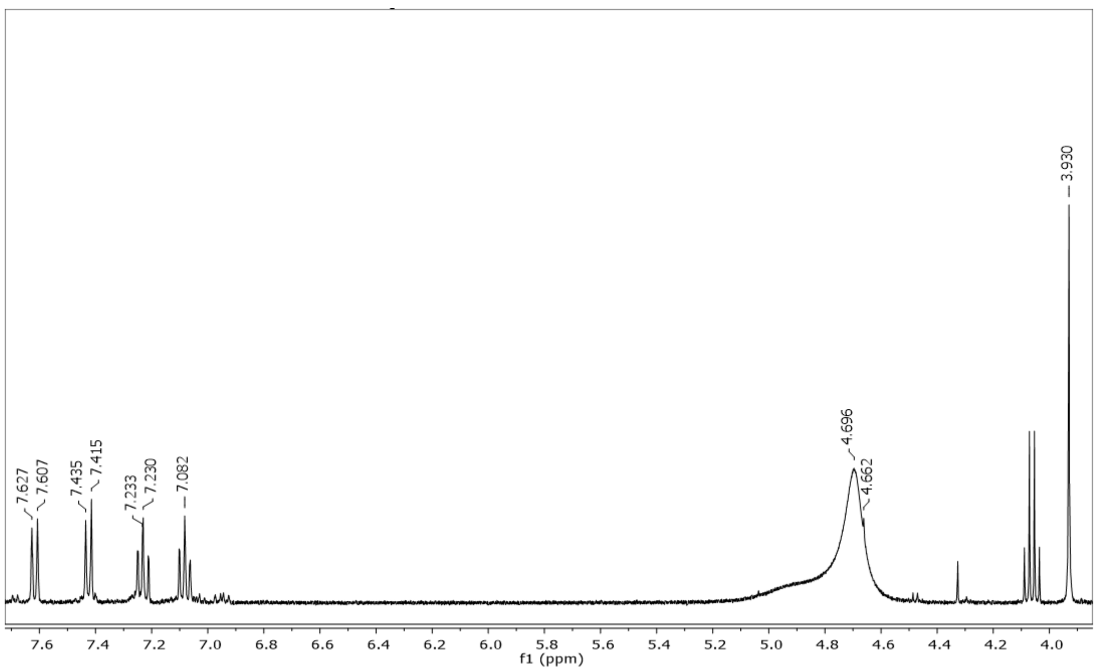

Figure S9. ^{1}H–NMR spectrum of IAA-SO_3H in CD_3OD/D_2O 2:1

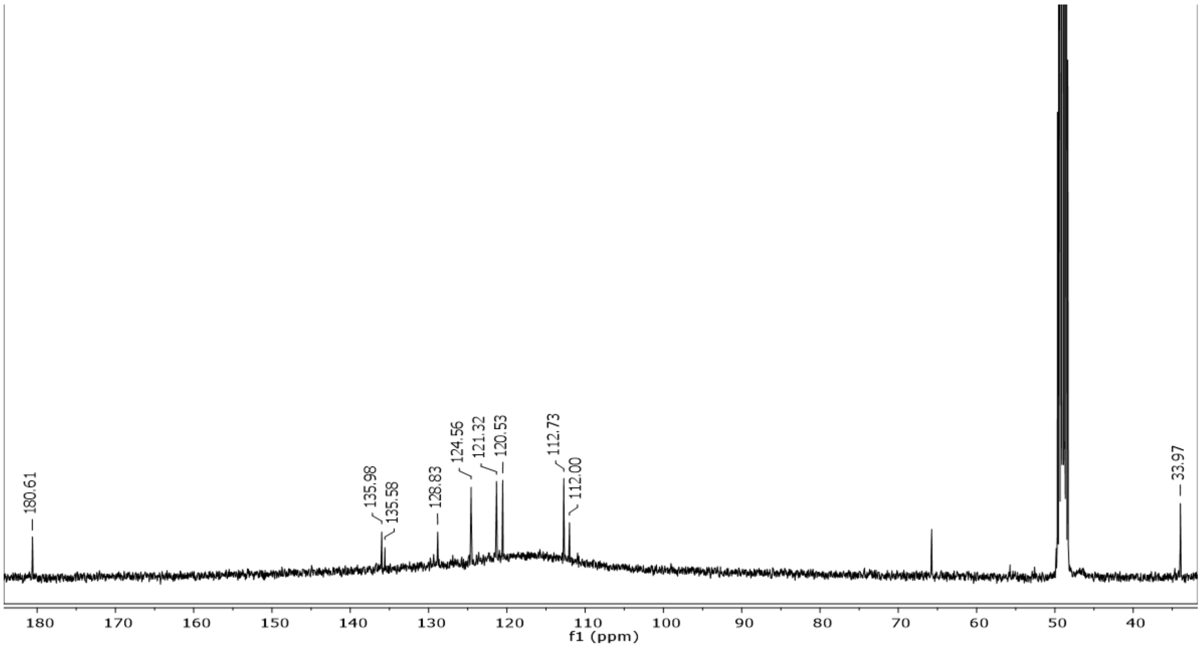

Figure S10. ^{13}C–NMR spectrum of IAA-SO_3H in CD_3OD/D_2O 2:1

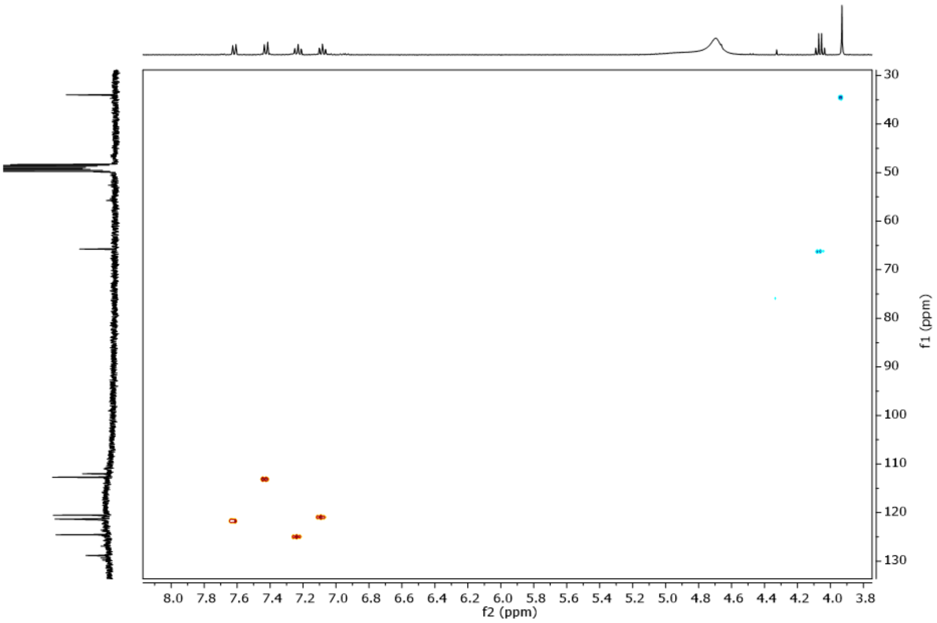

Figure S11. HSQC 2D-NMR spectrum of IAA-SO_3H in CD_3OD/D_2O 2:1

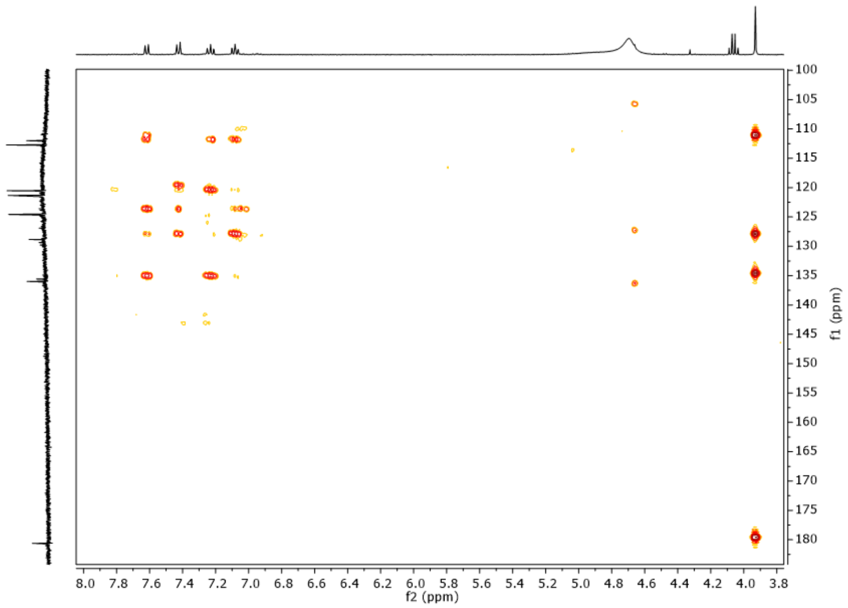

Figure S12. HMBC 2D-NMR spectrum of IAA-SO_3H in CD_3OD/D_2O 2:1

	Red wine				Rosé	Sparkling			White wines		
	1986-1998	2000-2008	2009-2013	2014-2015			Riserva	Rosé	2002-2009	2010-2014	2015-2016
**TOL**	0,11	4,65	11,92	19,89	50,41	1,15	0,53	0,87	3,33	6,05	1,09
**TOL-SO3H**	6,13	9,17	10,15	9,48	17,57	6,52	5,01	5,02	7,52	10,65	12,80
**ILA**	0,61	4,60	6,88	42,53	9,81	6,66	5,01	6,84	11,24	3,30	2,52
**ILA-SO3H**	17,25	3,34	2,61	6,81	8,78	9,42	11,39	7,73	15,88	6,12	10,68
**IAA**	56,14	0,00	0,22	0,13	42,99	0,00	0,00	0,00	0,24	0,28	0,00
**IAA-SO3H**	0,00	0,00	0,00	0,00	0,00	19,51	22,58	0,00	19,62	19,42	18,88
**ECAT**	1,00	21,23	21,51	38,49	6,47	0,00	0,00	0,98	0,00	3,38	6,94
**ECAT-SO3H**	27,17	50,70	10,54	1,20	10,39	0,00	0,00	0,00	0,00	0,00	0,00
**PROC-B**	0,88	19,34	22,91	40,65	9,97	0,00	0,00	0,00	0,00	0,89	5,35
**PROC-B-SO3H**	3,72	30,89	15,50	33,96	12,80	0,23	0,22	0,27	0,29	1,84	0,28
**KYN**	8,75	6,02	9,46	10,13	12,96	8,30	7,59	2,97	8,72	13,14	11,96
**KYNA**	21,57	11,88	16,80	10,52	12,13	3,50	4,61	3,97	6,52	4,35	4,13
**TRP**	0,36	3,30	4,58	27,31	26,31	0,66	0,25	0,09	3,58	20,41	13,16
**TRP-EE**	0,00	3,63	4,28	43,89	24,24	0,23	0,00	0,00	3,55	14,26	5,93
**N-TRP-EE**	5,11	15,29	17,78	19,08	12,03	4,31	3,46	3,79	6,93	6,86	5,36

Figure S13. Mean values (Supplementary Table S3) including data bar graph with the trend of principal metabolites in the various wine groups (green: free/sulfonated indoles; red: free/sulfonated flavanols; orange: KYN/KYNA; purple: tryptophan esters).

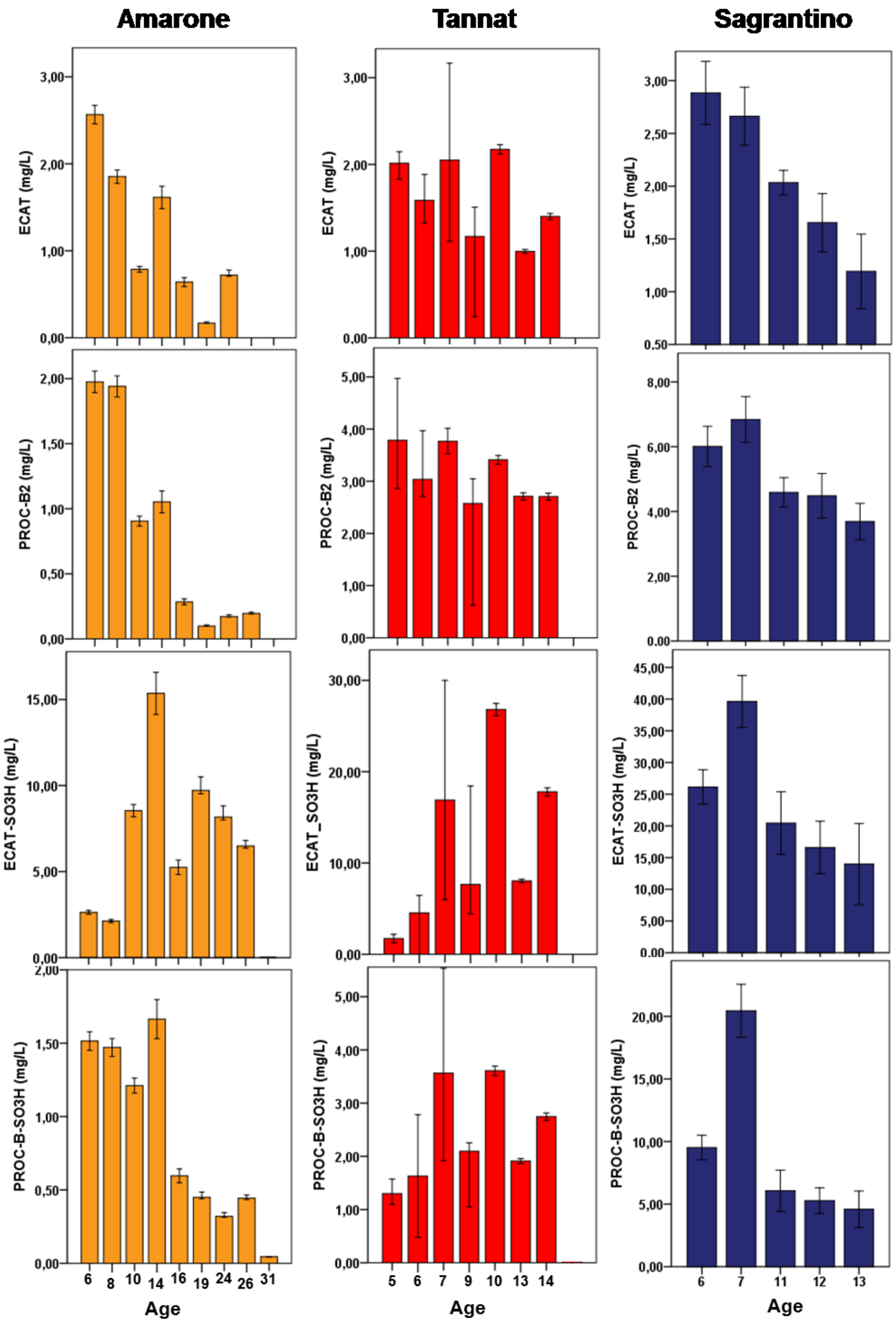

Figure S14. Behaviour of the unsulfonated ECAT and PROC-B2, and the sulfonated ECAT-SO3H and PROC-B-SO3H in relation wine age for the red wines Amarone (orange), Tannat (red) and Sagrantino (blue). All Amarone wines were produced by the same winery. Error bars represent a 95% confidence interval and the full data set is in Supplementary Table S3.

	Wine age													
	1	2	3	4	6	7	8	9	11	12	13	14	15	16
ILA	0,029	0,049	0,047	0,035	0,013	0,021	0,024	0,108	0,067	0,177	0,090	0,084	0,092	0,091
ILA-GLU	0,037	0,075	0,069	0,052	0,039	0,037	0,039	0,058	0,069	0,037	0,049	0,029	0,053	0,031
ILA-SO_3H	3,156	1,942	2,280	2,436	3,329	2,074	5,960	2,900	2,978	6,268	4,022	8,948	6,641	9,860
IAA-ASP	0,003	0,009	0,009	0,005	0,002	0,003	0,003	0,004	0,005	0,004	n.d.	0,001	0,002	n.d.
IPA	0,156	0,162	0,168	0,190	0,195	0,212	0,203	0,193	0,217	0,229	0,268	0,203	0,299	0,295
TRP	0,455	0,924	0,538	0,285	0,151	0,229	0,091	0,030	0,053	0,050	0,008	0,044	0,058	0,085
TRP-EE	0,029	0,052	0,033	0,020	0,016	0,021	0,014	0,006	0,007	0,004	n.d.	0,003	0,003	0,005
KYNA	0,031	0,038	0,042	0,048	0,046	0,063	0,056	0,057	0,056	0,068	0,085	0,074	0,088	0,075
KYN	0,009	0,004	0,003	0,006	0,005	0,006	0,006	0,002	0,005	0,002	0,001	0,002	0,003	0,003
TOL	0,111	0,182	0,144	0,117	0,088	0,120	0,069	0,108	0,081	0,084	0,060	0,050	0,076	0,050
TOL-SO_3H	2,254	1,860	0,807	1,160	2,103	1,388	2,273	0,332	0,815	0,668	0,851	0,628	1,354	0,859
TYR	2,017	2,168	2,502	2,148	1,723	1,967	1,660	2,789	2,529	2,101	3,097	3,049	2,668	3,099
N-TYR-EE	0,003	0,002	0,003	0,003	0,003	0,003	0,003	0,002	0,003	0,003	0,004	0,003	0,004	0,003
TYL	3,084	3,090	3,059	2,904	3,436	3,289	3,465	1,866	2,691	2,375	2,473	2,272	2,833	2,409
PHE	0,820	0,881	0,864	0,803	0,708	0,773	0,728	0,861	0,882	0,940	0,926	1,005	0,902	0,950
ABA-GLU	0,022	0,027	0,022	0,024	0,021	0,029	0,005	0,003	0,005	0,004	0,007	0,004	n.d.	n.d.
PROC-B-SO_3H	0,487	0,640	0,311	0,229	0,117	n.d.	0,056	n.d.	0,048	n.d.	n.d.	n.d.	0,049	n.d.
CAT	0,367	0,463	0,427	0,232	0,294	0,040	0,111	0,082	0,487	0,124	0,129	0,043	0,457	0,140
ECAT	0,675	1,022	0,581	0,278	0,226	n.d.	n.d.	n.d.	0,194	0,009	n.d.	n.d.	0,227	n.d.
PROC-B1	5,466	4,842	1,558	0,497	0,134	n.d.	n.d.	n.d.	0,082	n.d.	n.d.	n.d.	n.d.	n.d.
PROC-B2	0,771	0,959	0,391	0,151	0,047	n.d.	n.d.	n.d.	n.d.	n.d.	n.d.	n.d.	n.d.	n.d.

Figure S15. Verdicchio white wines metabolites mean values in mg/L (Supplementary Table S3) including data bar graph. Blue bars are used for the metabolites with a positive correlation with wine age and red bars for the metabolites with a negative correlation (Supplementary Table 5).

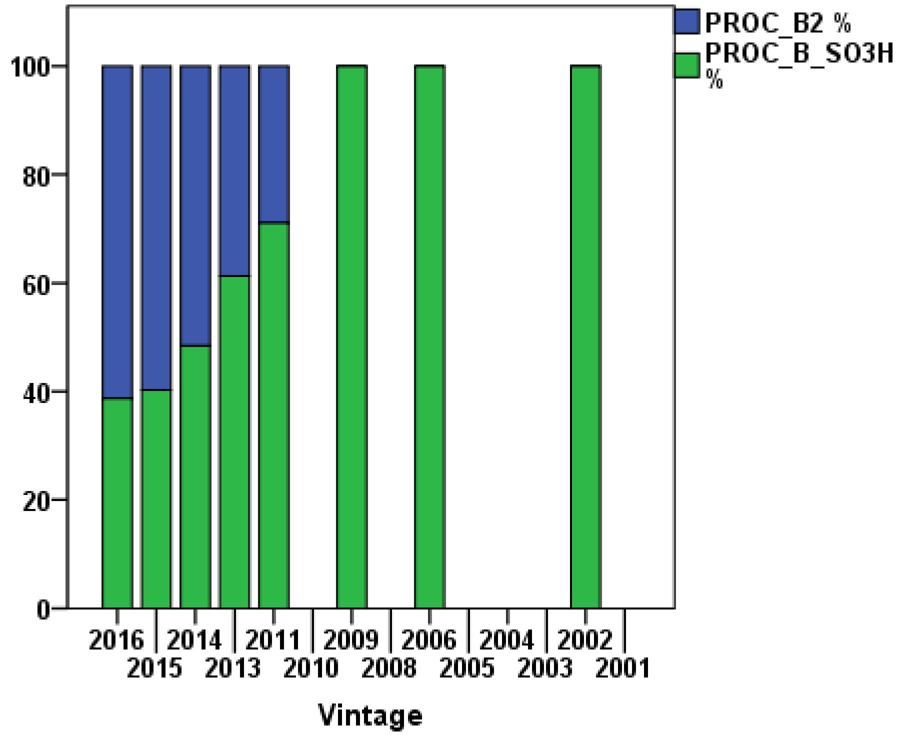

Figure S16. Comparison of the PROC-B2/PROC-B-SO3H relative percentage concentration for Verdicchio white wines in relation with the vintage/age.

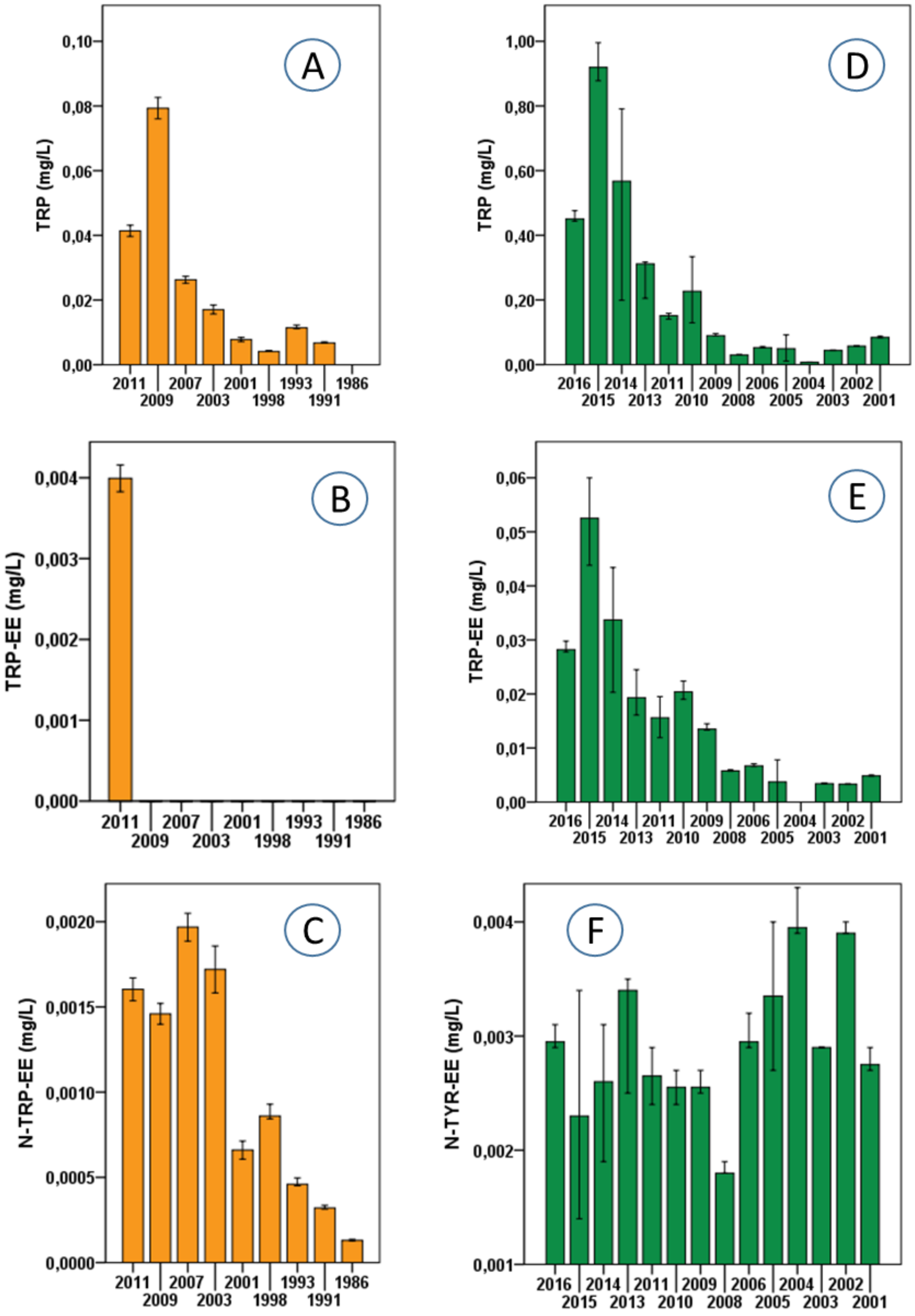

Figure S17. Concentration of TRP, TRP-EE and N-TRP-EE for the red wines of Amarone (A, B and C) and the white wines of Verdicchio (D, E and F) in relation the vintage. Error bars represent a 95% confidence interval and the full data set is in Supplementary Table S3.

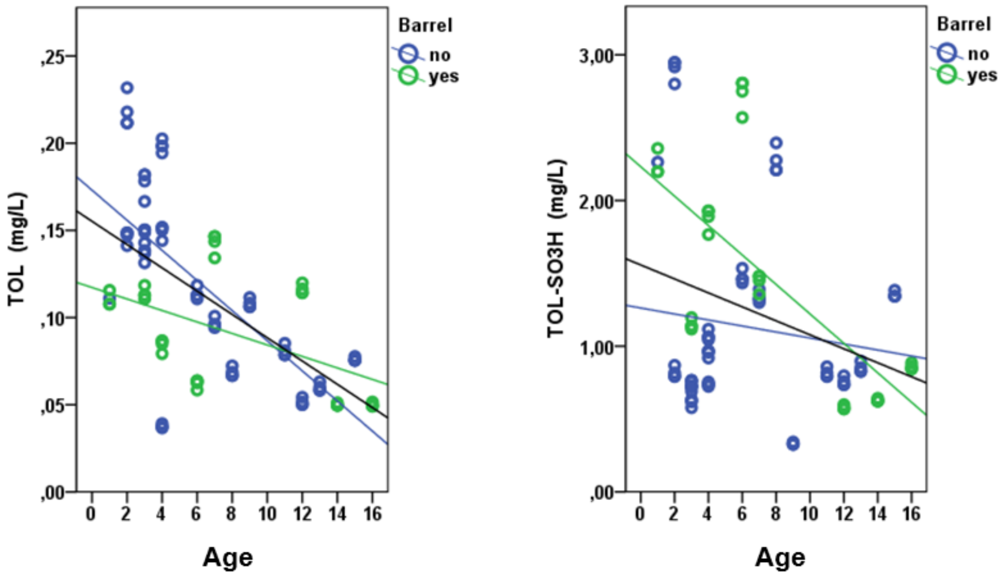

Figure S18. Concentration of TOL and TOL-SO3H for the white wines of Verdicchio in relation the wine age.

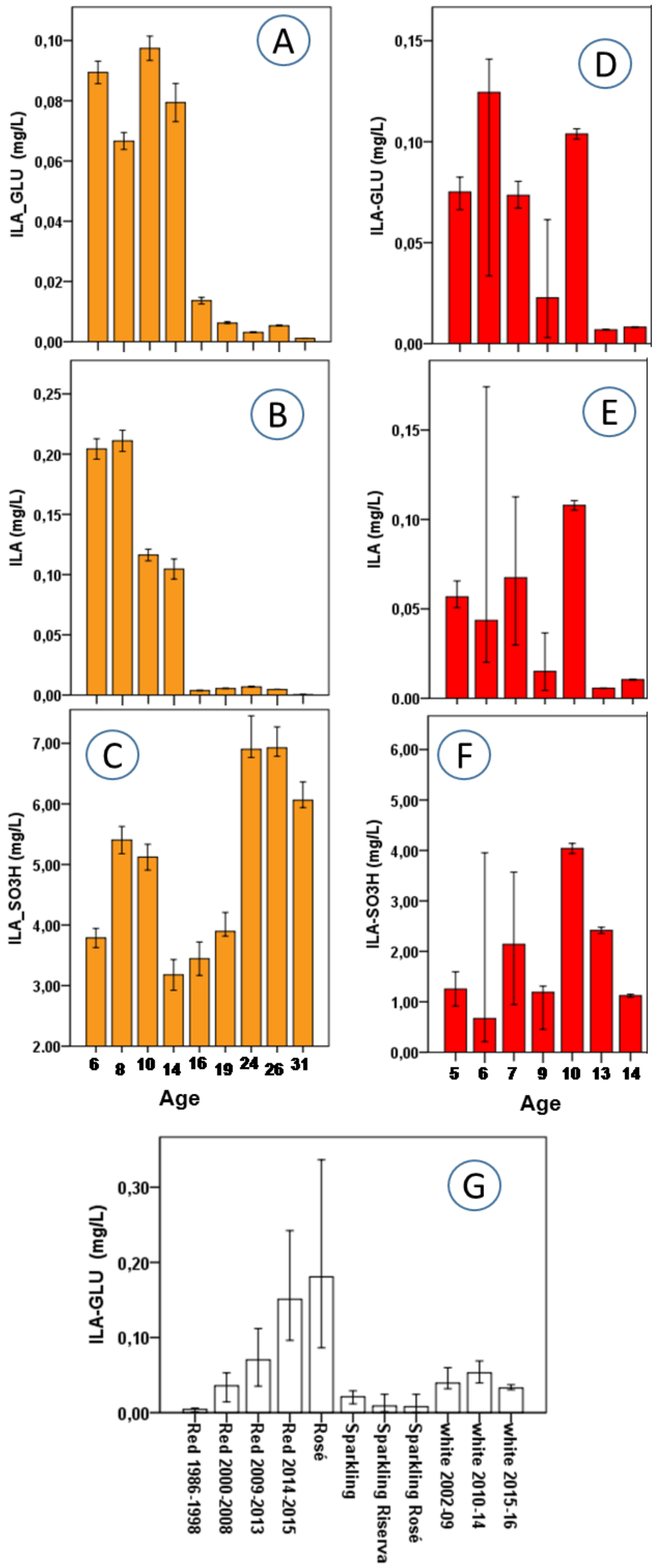

Figure S19. Concentration of ILA-GLU, ILA and ILA-SO3H for the red wines of Amarone (A, B and C) and Tannat (D, E and F) in relation the vintage. Graph G reports the concentration of ILA-GLU for various classes of wine. Error bars represent a 95% confidence interval and the full data set is in Supplementary Table S3.

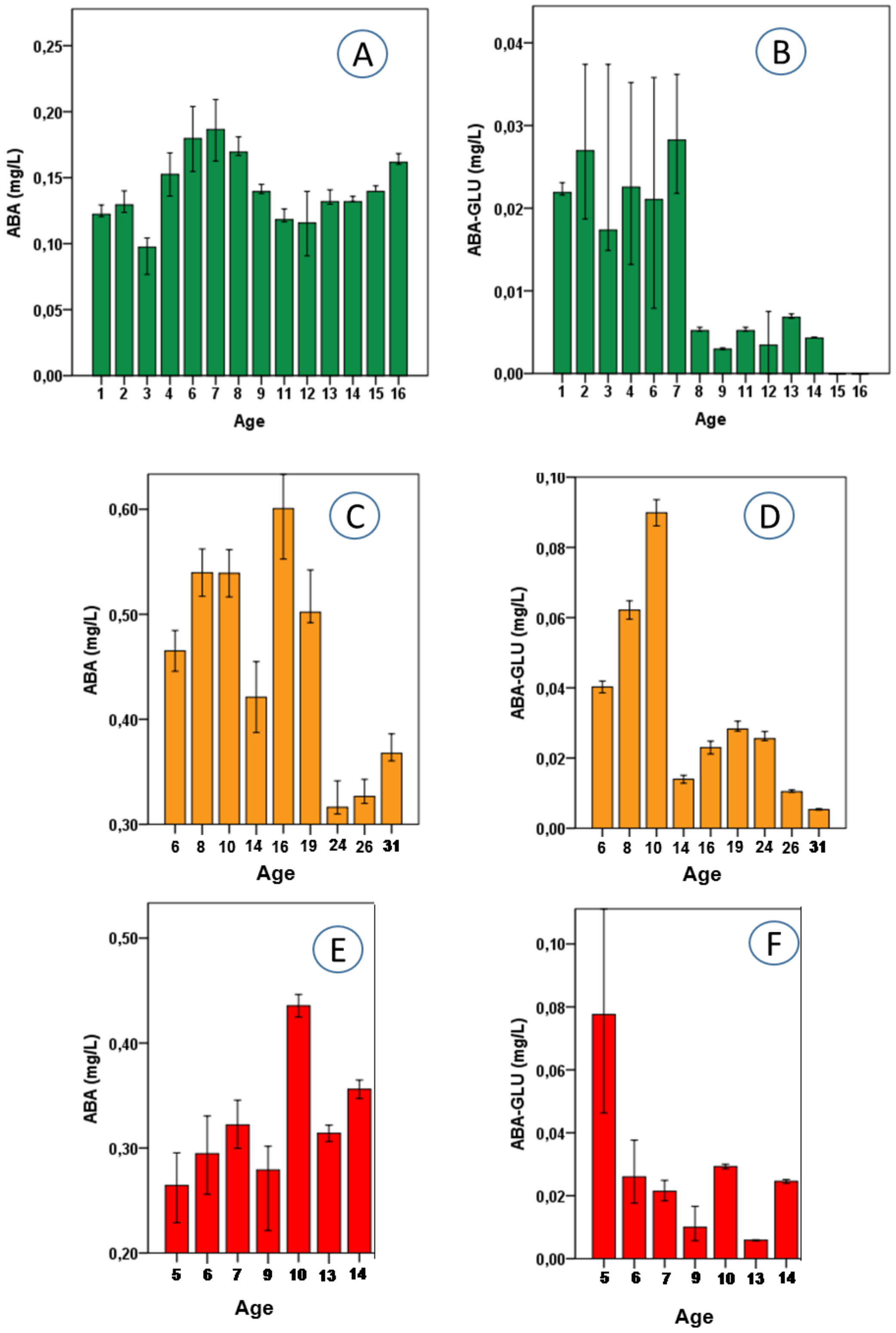

Figure S20. Concentration of ABA and ABA-GLU white wines Verdicchio (A-B), and red wines Amarone (C-D) and Tannat (E-F) in relation the wine age. Error bars represent a 95% confidence interval and the full data set is in Supplementary Table S3.
